# Efficiency and safety of varying the frequency of whole blood donation (INTERVAL): a randomised trial of 45 000 donors

**DOI:** 10.1016/S0140-6736(17)31928-1

**Published:** 2017-11-25

**Authors:** Emanuele Di Angelantonio, Simon G Thompson, Stephen Kaptoge, Carmel Moore, Matthew Walker, Jane Armitage, Willem H Ouwehand, David J Roberts, John Danesh, INTERVAL Trial Group, Emanuele Di Angelantonio, Simon G Thompson, Stephen Kaptoge, Carmel Moore, Matthew Walker, Jane Armitage, Willem H Ouwehand, David J Roberts, John Danesh, Jane Armitage, John Danesh, Emanuele Di Angelantonio, Jenny Donovan, Ian Ford, Rachel Henry, Beverley J Hunt, Bridget Le Huray, Susan Mehenny, Gail Miflin, Carmel Moore, Willem H Ouwehand, Jane Green, David J Roberts, Mike Stredder, Simon G Thompson, Matthew Walker, Nicholas A Watkins, Alan McDermott, Clive Ronaldson, Claire Thomson, Zoe Tolkien, Lorna Williamson, David Allen, John Danesh, Emanuele Di Angelantonio, Rachel Henry, Susan Mehenny, Carmel Moore, Willem H Ouwehand, David J Roberts, Jennifer Sambrook, Matthew Walker, Tracey Hammerton, Claire Thomson, Zoe Tolkien, David Allen, David Bruce, Fizzah Choudry, Emanuele Di Angelantonio, Cedric Ghevaert, Kirstie Johnston, Anne Kelly, Andrew King, Susan Mehenny, Gail Miflin, Alfred Mo, Carmel Moore, Willem H Ouwehand, Lizanne Page, Penny Richardson, David J Roberts, Jennifer Sambrook, Peter Senior, Yagnesh Umrania, Matthew Walker, Henna Wong, Stephen Kaptoge, Gavin Murphy, Adrian C Newland, Keith Wheatley, Michael Greaves, Marc Turner, Tahir Aziz, Richard Brain, Christine Davies, Ruth Turner, Paula Wakeman, Alison Dent, Alan Wakeman, Ben Anthony, Desmond Bland, Will Parrondo, Helen Vincent, Candy Weatherill, Andrea Forsyth, Carol Butterfield, Tracey Wright, Karen Ellis, Kirstie Johnston, Pat Poynton, Carolyn Brooks, Emma Martin, Lara Littler, Lindsay Williams, Donna Blair, Karen Ackerley, Lynn Woods, Sophie Stanley, Gemma Walsh, Gayle Franklin, Cheryl Howath, Sarah Sharpe, Deborah Smith, Lauren Botham, Caroline Williams, Claire Alexander, Gareth Sowerbutts, Diane Furnival, Michael Thake, Shilpa Patel, Carolyn Roost, Sandra Sowerby, Mary Joy Appleton, Eileen Bays, Geoff Bowyer, Steven Clarkson, Stuart Halson, Kate Holmes, Gareth Humphries, Kirstie Johnston, Lee Parvin-Cooper, Jason Towler, Joanne Addy, Patricia Barrass, Louise Stennett, Susan Burton, Hannah Dingwall, Rachel Henry, Victoria Clarke, Maria Potton, Claire Thomson, Thomas Bolton, Michael Daynes, Stuart Halson, Sarah Spackman, Matthew Walker, Abudu Momodu, James Fenton, Adam King, Omer Muhammed, Nicholas Oates, Tim Peakman, Christine Ryan, Kristian Spreckley, Craig Stubbins, Joanna Williams, James Brennan, Cedric Mochon, Samantha Taylor, Kimberley Warren, Stephen Kaptoge, Simon G Thompson, Emanuele Di Angelantonio, Carmel Moore, Jonathan Mant, Willem H Ouwehand, Simon G Thompson, John Danesh, David J Roberts

**Affiliations:** a NHS Blood and Transplant, Cambridge, UK; b NHS Blood and Transplant, Oxford, UK; c Department of Public Health and Primary Care, Strangeways Research Laboratory, Cambridge, UK; d NIHR Blood and Transplant Research Unit in Donor Health and Genomics, Strangeways Research Laboratory, Cambridge, UK; e NIHR Cambridge Biomedical Research Centre, Addenbrooke's Hospital, Cambridge, UK; f British Heart Foundation Cambridge Centre for Research Excellence, Addenbrooke's Hospital, Cambridge, UK; g Department of Haematology, University of Cambridge, Cambridge Biomedical Campus, Cambridge, UK; h NIHR Oxford Biomedical Research Centre—Haematology Theme and Radcliffe Department of Medicine, University of Oxford, John Radcliffe Hospital, Oxford, UK; i Clinical Trial Service Unit and Epidemiological Studies Unit, Nuffield Department of Population Health, University of Oxford, Oxford, UK; j MRC Population Health Research Unit, University of Oxford, Oxford, UK

## Abstract

**Background:**

Limits on the frequency of whole blood donation exist primarily to safeguard donor health. However, there is substantial variation across blood services in the maximum frequency of donations allowed. We compared standard practice in the UK with shorter inter-donation intervals used in other countries.

**Methods:**

In this parallel group, pragmatic, randomised trial, we recruited whole blood donors aged 18 years or older from 25 centres across England, UK. By use of a computer-based algorithm, men were randomly assigned (1:1:1) to 12-week (standard) versus 10-week versus 8-week inter-donation intervals, and women were randomly assigned (1:1:1) to 16-week (standard) versus 14-week versus 12-week intervals. Participants were not masked to their allocated intervention group. The primary outcome was the number of donations over 2 years. Secondary outcomes related to safety were quality of life, symptoms potentially related to donation, physical activity, cognitive function, haemoglobin and ferritin concentrations, and deferrals because of low haemoglobin. This trial is registered with ISRCTN, number ISRCTN24760606, and is ongoing but no longer recruiting participants.

**Findings:**

45 263 whole blood donors (22 466 men, 22 797 women) were recruited between June 11, 2012, and June 15, 2014. Data were analysed for 45 042 (99·5%) participants. Men were randomly assigned to the 12-week (n=7452) versus 10-week (n=7449) versus 8-week (n=7456) groups; and women to the 16-week (n=7550) versus 14-week (n=7567) versus 12-week (n=7568) groups. In men, compared with the 12-week group, the mean amount of blood collected per donor over 2 years increased by 1·69 units (95% CI 1·59–1·80; approximately 795 mL) in the 8-week group and by 0·79 units (0·69–0·88; approximately 370 mL) in the 10-week group (p<0·0001 for both). In women, compared with the 16-week group, it increased by 0·84 units (95% CI 0·76–0·91; approximately 395 mL) in the 12-week group and by 0·46 units (0·39–0·53; approximately 215 mL) in the 14-week group (p<0·0001 for both). No significant differences were observed in quality of life, physical activity, or cognitive function across randomised groups. However, more frequent donation resulted in more donation-related symptoms (eg, tiredness, breathlessness, feeling faint, dizziness, and restless legs, especially among men [for all listed symptoms]), lower mean haemoglobin and ferritin concentrations, and more deferrals for low haemoglobin (p<0·0001 for each) than those observed in the standard frequency groups.

**Interpretation:**

Over 2 years, more frequent donation than is standard practice in the UK collected substantially more blood without having a major effect on donors' quality of life, physical activity, or cognitive function, but resulted in more donation-related symptoms, deferrals, and iron deficiency.

**Funding:**

NHS Blood and Transplant, National Institute for Health Research, UK Medical Research Council, and British Heart Foundation.

## Introduction

Worldwide, tens of millions of whole blood donors provide around 110 million donations annually, enabling life-saving transfusions for many clinical indications.^1^ Yet, despite more than a century of blood donation, the efficiency and safety of different approaches to blood collection have not been properly evaluated. In particular, no randomised trial has yet investigated the effect of different inter-donation intervals on blood supply and donor health. The absence of evidence has resulted in widely varying policies and practices across international blood services, producing uncertain and, potentially, non-optimal outcomes for blood donors. For example, in the UK, the current practice is to allow men to donate every 12 weeks and women every 16 weeks.^2^ By contrast, in the USA, men and women can donate every 8 weeks. In France and Germany, men can donate every 8 weeks and women every 12 weeks.^3^, ^4^

In recent years, demand for blood has generally declined in western countries, probably because of adoption of lower haemoglobin triggers for transfusion and other approaches to avoid transfusion.^5^, ^6^ However, there is growing demand for universal blood groups (eg, O Rhesus D [RhD] negative and A RhD negative) and for minor blood groups that might be needed to support multiply transfused populations (eg, patients with sickle cell disease).^5^ In the longer term, the decline in demand could slow (or even reverse) as a result of population ageing. In parallel, maintenance of the blood supply could become more difficult than at present as blood services encounter problems in attracting and retaining young donors.^7^

**Research in context**

**Evidence before this study**

We searched for randomised trials published in English before March 1, 2017, investigating the effect of varying the whole blood inter-donation interval. We searched PubMed, Scientific Citation Index Expanded, and Embase using relevant terms: “blood donation intervals”, “blood donation frequency”, “blood supply”, and “donor health”. Although blood donation has been practised for more than 100 years, we could not identify any randomised trials on this topic.

**Added value of this study**

As the first-ever such randomised trial, this study should provide uniquely reliable insight into the consequences of reducing the inter-donation interval. Furthermore, because the trial was embedded in a national blood service, it had major additional advantages. First, it recorded a comprehensive range of outcomes related to donation efficiency, safety, and biochemistry. Second, it achieved a clear separation across randomised groups because of good adherence to the trial interventions. Third, it achieved rapid recruitment across the geographical breadth of England, UK, and included participants broadly representative of the national donor population. Fourth, it randomised more than 45 000 participants, providing excellent statistical power. Fifth, it achieved 99·5% completeness in the primary outcome.

**Implications of all the available evidence**

Our data give policy makers in the UK the short-term option of allowing more frequent collection from donors than is now standard, such as for in-demand blood groups or during periods of falling supply. Our data also quantify the extent of iron depletion within 2 years of repeated donation, thus informing safety guidelines. Finally, our results suggest a need to use comprehensive reminders to help donors make and keep appointments, and to review the screening method used in the UK to test individuals' eligibility to donate.

One approach to managing the blood supply is to collect blood more frequently from existing donors.^8^ However, limits on the frequency of donation exist to safeguard donor health and the quality of blood components. In observational studies, shorter than average inter-donation intervals have been associated with higher frequency of iron deficiency, lower haemoglobin, and higher rates of deferral (temporary suspension of donors from giving blood) because of failure of donors to meet minimum haemoglobin concentrations.^9^, ^10^, ^11^ However, such studies have been liable to biases and have generally failed to collect information systematically on relevant outcomes.

We report the results of the INTERVAL trial, which aimed to assess the effect of different inter-donation intervals on blood supply and donor health over a 2-year period.

## Methods

### Trial design and participants

INTERVAL was a large, parallel group, pragmatic, randomised trial. Full details of the trial's objectives, design, and recruitment have been published.^12^, ^13^ Eligible donors were aged 18 years or older, fulfilled routine criteria for donation, had an email address and access to the internet to respond to web-based questionnaires, and were willing to be randomly assigned to any of the trial's intervention groups at one of the 25 static donor centres of NHS Blood and Transplant (NHSBT), the sole blood provider to the National Health Service (NHS) in England, UK. Donors underwent routine screening for eligibility, including haemoglobin screening via a gravimetric method (copper sulphate test), followed by the spectrophotometric HemoCue test (HemoCue AB, Ängelholm, Sweden) with venous blood for those who failed the copper sulphate test (minimum thresholds to donate in England are 135 g/L for men and 125 g/L for women).^14^ If, for any reason, the donor was not eligible to make a donation on that day, they could not join the trial on that occasion. After reading study leaflets and participating in a discussion with donor carer staff, eligible donors were asked to complete the trial consent form before giving a blood donation. The National Research Ethics Service approved (11/EE/0538) this study.

### Randomisation and blinding

Men were randomly assigned to 12-week (standard) versus 10-week versus 8-week inter-donation intervals, and women to 16-week (standard) versus 14-week versus 12-week intervals, without any other limit on the maximum number of donations allowed during the study period. Balanced randomisation of donors to sex-specific intervention groups in the ratio of 1:1:1 was done at the coordinating centre by use of a computer program built into the trial database, with a minimisation algorithm to ensure key characteristics (age, weight, and numbers of new *vs* existing donors) were balanced across trial groups at baseline. Randomisation was stratified by donation centre. Because of the nature of the intervention, it was not possible to blind participants to their allocated intervention group.

### Procedures

Donors were recruited between June 11, 2012, and June 15, 2014.^13^ Immediately after enrolment, participants received online questionnaires, including the 36-item Short Form Health Survey, version 2 (SF-36v2).^15^ Only participants who completed baseline questionnaires were eligible for randomisation. Participants were advised of their allocated inter-donation interval by email, which was recorded in the NHSBT donor database. A non-fasting research blood sample was taken before donation at the enrolment visit and at the 2-year survey (but not at the intervening donations) and transported to a central laboratory for a full blood count analysis (Sysmex XN-2000 haematology analyser, UK BioCentre, Stockport, UK). Aliquots of EDTA (edetic acid) plasma, serum, and buffy coat were stored at −80°C. DNA was extracted from buffy coat by use of a Kleargene method (LGC Genomics, Teddington, UK). Technicians unaware of the intervention groups to which participants had been allocated did laboratory assays. Samples of sufficient concentration and purity were aliquoted for shipment to Affymetrix (Santa Clara, CA, USA), where genotyping was done for haemochromatosis gene (*HFE)* mutation carrier status as part of a high-density genotyping array (Biobank Axiom Array, Santa Clara, CA, USA). Ferritin concentrations were measured at baseline and at the 2-year survey (with the 2-year survey only done in a randomly selected subset) in stored serum samples with an immunoturbidimetric assay (Roche/Hitachi chemistry analyser, Stichting Huisartsen Laboratorium, Etten-Leur, Netherlands). We regarded ferritin results as post-hoc findings, since they were not included in the prespecified analysis plan.

The INTERVAL trial used a more comprehensive approach to remind participants to make and keep donation appointments than that used routinely by NHSBT, including a uniform protocol of email, text message, and telephone reminders (appendix). At 6, 12, and 18 months of follow-up, donor safety characteristics were monitored by participants' online responses to the abbreviated 12-item Short Form Health Survey, version 2 (SF-12v2).^16^ At these timepoints, donors were asked about the presence of symptoms potentially related to blood donation that had occurred in the previous 6 months. At 2 years after randomisation, participants received an extended version of the same questionnaire asking about symptoms potentially related to blood donation, again focusing on the previous 6 months (apart from pica [a craving to eat non-food items], for which the relevant time period of queries related to the previous 2 years; appendix). At the 2-year survey, participants also received the SF-36v2, cognitive function tests (attention and reaction time, executive function, episodic memory, and intelligence),^17^ and a physical activity questionnaire (the Recent Physical Activity Questionnaire).^18^ The deferral policy used in the trial was the same as that used in routine NHSBT practice (appendix). For example, male donors with measured haemoglobin concentrations of 125–134 g/L and female donors with concentrations of 115–124 g/L were deferred for a period of 3 months. At a participant's last donation before completing the 2-year involvement in the trial, blood research sampling was repeated as described above. If participants were deferred or medically withdrawn from blood donation at that time, they gave a research sample only.

### Outcomes

The primary outcome was the number of blood donations over 2 years, with standard practice being to donate 1 unit of blood per session (full donation unit 470 mL). Secondary outcomes related to safety were quality of life, donation-related symptoms, physical activity, cognitive function, haemoglobin and ferritin concentrations, and deferrals because of low haemoglobin. Physical wellbeing at 2 years, assessed with the physical component score of SF-36v2, was prespecified as the key secondary outcome. We compared both the primary outcome and the key secondary outcome between each of the higher frequency groups versus the standard frequency group (for men: 8 *vs* 12 weeks and 10 *vs* 12 weeks; for women: 12 *vs* 16 weeks and 14 *vs* 16 weeks). For other outcomes and prespecified tests for interaction, linear trend was assessed across randomised groups. Certain secondary outcomes related to safety (eg, self-reported symptoms) involved a combination of data from multiple donation sessions attended, or multiple questionnaires answered, by each participant.

### Statistical analysis

The statistical analysis followed a prespecified plan (appendix). Briefly, data for men and women were analysed separately by the intention-to-treat principle according to their randomised groups.

Ferritin values were log_e_ transformed and presented as geometric means, and used to classify donors as iron depleted (<15 μg/L) according to WHO criteria.^19^ For all other outcomes, we present means and percentages without adjustment. We compared randomised groups by calculating p values for differences or linear trend using normal regression models for continuous outcomes and logistic regression models for binary outcomes adjusted for centre, age, weight, new donor status, and baseline value of the outcome (when relevant). Because of the number of statistical tests done, we used the following guidelines for considering whether the results provided strong evidence: p less than 0·005 for the main analysis of the number of donations over 2 years and physical component score at 2 years; p less than 0·0005 for their interaction tests; and p less than 0·0002 for the tests of trend for the other secondary outcomes.

The sample size calculation was based on having 80% power to detect a difference of 5% or more in donation rates (the primary outcome) in subgroups with a prevalence of 10% or more. This calculation assumed a type I error of 0·05 and mean donation rate of 1·6 times per year in the standard donation frequency group, and between-subject SD of 0·7 times per year in each group. Furthermore, the sample size was estimated to provide 80% power to detect a mean difference of 3% or more in the physical component score of the SF-36v2 (the key secondary outcome) in subgroups with a prevalence of 10% or more. This calculation assumed a type I error of 0·05 and mean physical component score of 50 in the standard donation frequency group, and between-subject SD of 10 in each group. Analyses were done with Stata, version 13.

This trial is registered with ISRCTN, number ISRCTN24760606.

### Role of the funding source

The academic investigators and representatives of NHSBT, a funder of the trial, participated in the study design and oversight. The investigators at the trial's academic coordinating centre had sole access to the trial database, and had final responsibility for data collection, data integrity, data analysis, and data interpretation, as well as manuscript drafting and the decision to submit the manuscript for publication. All authors gave approval to submit for publication.

## Results

**Figure 1:**
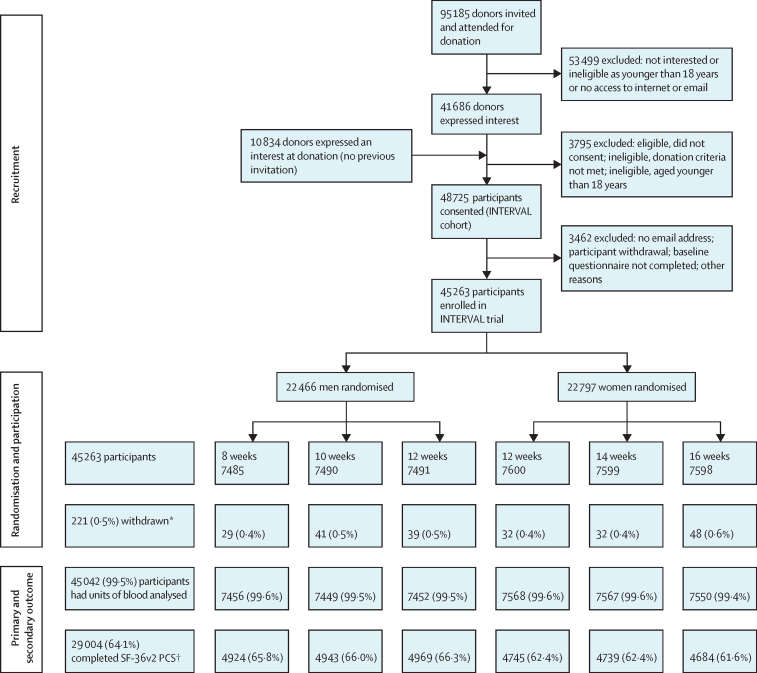
Trial profile CONSORT flowchart showing recruitment, participation, and completeness of main outcomes. *Participants withdrew permission to use their data. †Physical wellbeing at 2 years was measured with the physical component score (PCS) of the 36-item Short Form Health Survey, version 2 (SF-36v2).

45 263 donors (22 466 men, 22 797 women) were randomly assigned to different inter-donation intervals (figure 1; appendix). 221 (0·5%) participants withdrew permission to use their data. Baseline characteristics were balanced across randomised groups (table 1). Mean age was 45·3 years (SD 14·2) for men and 41·4 years (14·0) for women; mean weight was 85·1 kg (14·5) for men and 71·6 kg (14·8) for women. Mean baseline haemoglobin values, which were available for 44 148 (98%) participants, were 149·7 g/L (SD 10·0) for men and 133·9 g/L (9·2) for women. 1191 (5·4%) of 21 977 men who passed the routine NHSBT haemoglobin eligibility check were found to have venous concentrations less than 135 g/L and 3037 (13·7%) of 22 171 women were found to have venous concentrations less than 125 g/L with a haematology analyser. Geometric mean baseline ferritin values, which were available for 42 155 (94%) participants, were 44·9 μg/L (IQR 27·0–77·0) in men and 24·6 μg/L (15·0–44·0) in women. 1812 (8·6%) of 21 011 men and 5260 (24·9%) of 21 144 women had ferritin concentrations less than 15 μg/L. 1798 (8·0%) of 22 357 men and 2411 (10·6%) of 22 685 women were classified as new donors. The mean number of whole blood donations in the 2 years preceding the trial was 3·58 for men and 2·87 for women, with a deferral rate for low haemoglobin per session attended of 1·1% for men and 3·6% for women (table 1). As published previously, participants were broadly representative of the national donor population of England, with only minor differences in age, sex, and number of previous donations (appendix).^13^

**Table 1:** Baseline characteristics by sex and intervention group

	**Men**			**Women**		
	8 weeks	10 weeks	12 weeks	12 weeks	14 weeks	16 weeks
Number of participants*	7456	7449	7452	7568	7567	7550
Age, years	45·3 (14·2)	45·2 (14·2)	45·3 (14·2)	41·3 (14·0)	41·4 (13·9)	41·4 (14·0)
Weight, kg	85·1 (14·4)	85·1 (14·7)	85·1 (14·4)	71·3 (14·4)	71·8 (15·1)	71·8 (14·8)
SF-36v2 physical component score	56·8 (4·6)	56·9 (4·5)	56·8 (4·5)	57·0 (4·7)	57·0 (4·7)	57·0 (4·6)
SF-36v2 mental component score	54·6 (6·0)	54·5 (6·3)	54·5 (6·1)	53·5 (6·7)	53·5 (6·6)	53·5 (6·5)
Haemoglobin concentration, g/L	149·8 (10·1)	149·6 (9·9)	149·8 (9·9)	133·9 (9·4)	134·0 (9·1)	133·8 (9·0)
Haemoglobin concentration <135 g/L (men) or <125 g/L (women)	402 (5·5%)	404 (5·5%)	385 (5·3%)	1022 (13·8%)	1028 (13·9%)	987 (13·4%)
Ferritin concentration, μg/L†	45·3 (27·0–77·0)	44·1 (27·0–76·0)	45·4 (28·0–77·0)	24·9 (14·0–45·0)	24·5 (15·0–44·0)	24·4 (15·0–44·0)
Ferritin concentration <15 μg/L	614 (8·7%)	649 (9·3%)	549 (7·9%)	1769 (25·1%)	1730 (24·5%)	1761 (25·0%)
New donor‡	601 (8·1%)	598 (8·0%)	599 (8·0%)	808 (10·7%)	801 (10·6%)	802 (10·6%)
Number of blood donations in previous 2 years§	3·58 (1·87)	3·59 (1·86)	3·57 (1·85)	2·87 (1·67)	2·88 (1·65)	2·85 (1·66)
Deferral rate for low haemoglobin in previous 2 years¶	1·09%	1·03%	1·03%	3·58%	3·51%	3·70%

Data are mean (SD) or number of participants (%), unless otherwise stated.

* Excluding the 0·5% who withdrew permission to use their data. Additional missing data: none for age, weight, or donation history; 0·7% for 36-item Short Form Health Survey, version 2 (SF-36v2) physical component score, 0·7% for SF-36v2 mental component score, 2·0% for haemoglobin concentration, and 6·4% for ferritin concentration.

† Values are geometric means and IQRs.

‡ A participant who had not previously provided a full blood donation.

§ After excluding new donors, the mean number of whole blood donations in the 2 years preceding the trial was 3·94 for men and 3·20 for women.

¶ Deferral rate (%) per donation session attended averaged over all previous attendances in previous 2 years.

Median times between attending for donation were 12·3 weeks (IQR 12·0–15·4) for men allocated to the 12-week group, 10·1 weeks (10·0–13·0) for the 10-week group, and 8·3 weeks (8·0–12·0) for the 8-week group (appendix). Median times were 16·6 weeks (IQR 16·0–22·0) for women allocated to the 16-week group, 14·3 weeks (14·0–19·0) for the 14-week group, and 12·7 weeks (12·0–17·3) for the 12-week group. For men, more than 70% of attendances were achieved within 2 weeks of the interval allocated. For women, more than 70% of attendances were achieved within 3 weeks of the interval allocated.

**Figure 2:**
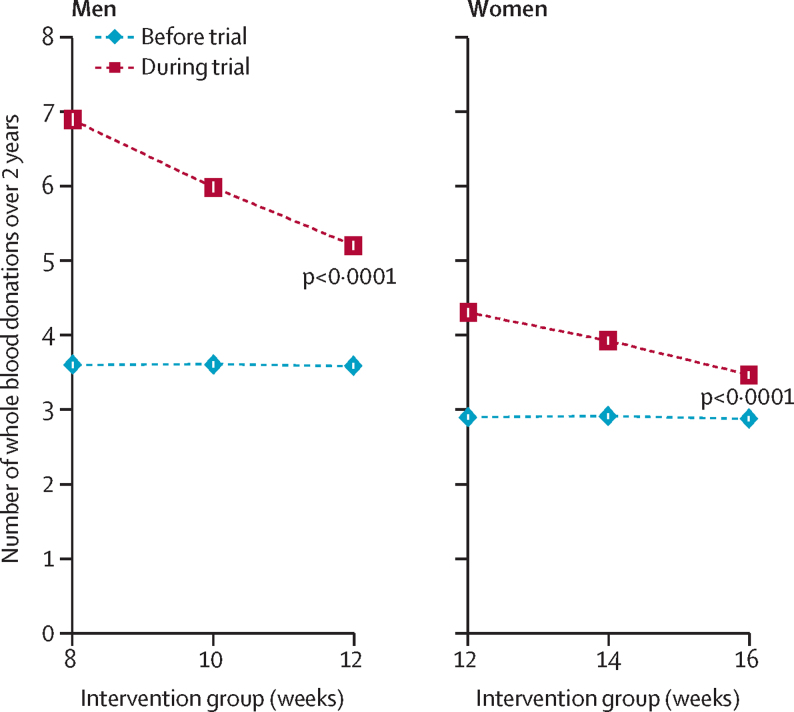
Number of whole blood donations during the 2-year trial period and in the previous 2 years by sex and intervention group The p values compare randomised groups (shown in red) adjusted for baseline characteristics (centre, age, weight, and new donor status). Mean (95% CI) numbers of whole blood donations made by the same individuals in the 2 years before the trial (shown in blue) are provided for context. Minimum inter-donation intervals allowed before the trial were 12 weeks for men and 16 weeks for women.

Information on the primary outcome was available for 45 042 (99·5%) participants. In men, compared with the standard 12-week group, the mean amount of blood collected per donor increased by 1·69 units (95% CI 1·59–1·80; approximately 795 mL) in the 8-week group and by 0·79 units (0·69–0·88; approximately 370 mL) in the 10-week group (p<0·0001 for both; table 2; figure 2). In women, compared with the standard 16-week group, the mean amount of blood collected per donor increased by 0·84 units (95% CI 0·76–0·91; approximately 395 mL) in the 12-week group and 0·46 units (0·39–0·53; approximately 215 mL) in the 14-week group (p<0·0001 for both; table 2; figure 2). Among donors with at least a 2-year history of blood donation before trial enrolment, the mean number of donations during the trial was 38% higher (5·40 *vs* 3·91 units; appendix) than in the preceding 2 years for men allocated to the 12-week group, and 15% higher (3·63 *vs* 3·17 units; appendix) for women allocated to the 16-week group.

**Table 2:** Number of whole blood donations over 2 years and physical component score at 2 years by sex and intervention group

	**Men**			**Women**		
	8 weeks	10 weeks	12 weeks	12 weeks	14 weeks	16 weeks
**Whole blood donations over 2 years**						
Mean number (95% CI)	6·88 (6·80 to 6·97)	5·97 (5·90 to 6·05)	5·19 (5·13 to 5·25)	4·28 (4·22 to 4·34)	3·90 (3·85 to 3·96)	3·44 (3·40 to 3·49)
Mean difference (95% CI)	1·69 (1·59 to 1·80)	0·79 (0·69 to 0·88)	Reference group	0·84 (0·76 to 0·91)	0·46 (0·39 to 0·53)	Reference group
p value*	<0·0001	<0·0001	··	<0·0001	<0·0001	··
**SF-36v2 physical component**†**score at 2 years**						
Mean score (95% CI)	56·5 (56·4 to 56·6)	56·6 (56·5 to 56·7)	56·5 (56·4 to 56·7)	56·4 (56·3 to 56·6)	56·5 (56·4 to 56·6)	56·3 (56·2 to 56·4)
Mean difference (95% CI)	−0·00 (−0·18 to 0·18)	0·06 (−0·12 to 0·23)	Reference group	0·14 (−0·06 to 0·33)	0·20 (0·01 to 0·40)	Reference group
p value*	0·996	0·438	··	0·304	0·048	··

Missing data (beyond the 0·5% who withdrew permission to use their data): none for number of donations; 35·6% for 2-year physical component score. SF-36v2=36-item Short Form Health Survey, version 2.

* p values are from analyses adjusted for baseline characteristics (centre, age, weight, and new donor status) and baseline physical component score for 2-year physical component score.

† Higher physical component scores indicate better physical wellbeing (0–100 scale range).

The mean physical component score at 2 years (for which data were available for 29 004 [64·3%] of 45 042 participants; appendix) did not differ across randomised groups (table 2; appendix). Similarly, there were no differences in a mixed model analysis incorporating physical component score data from all 6-monthly questionnaires, which involved data from 38 683 (85·9%) of 45 042 participants (all p>0·005). There were no major differences across randomised groups in the availability of physical component score data for 6-monthly or 2-year questionnaires.

**Figure 3:**
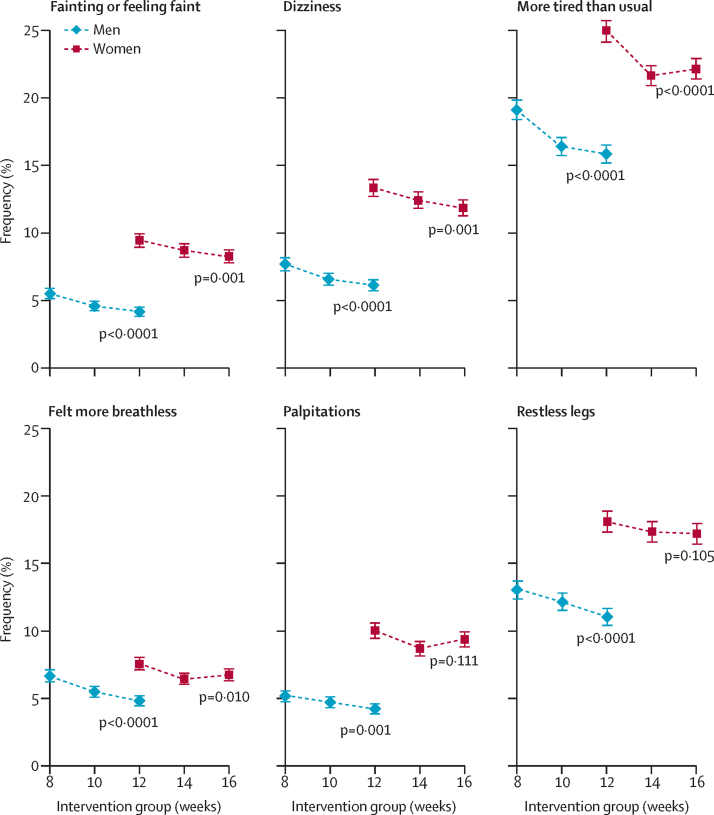
Self-reported symptoms during the 2-year trial period by sex and intervention group The p values assess trends across randomised groups, adjusted for baseline characteristics (centre, age, weight, and new donor status).

The availability of data for additional self-reported outcomes did not differ across randomised groups (appendix). There was no evidence of trends across randomised groups in the mental component score of the SF-36v2, cognitive function, or physical activity (table 3; appendix). Similarly, in the groups randomly assigned to higher donation frequencies than the standard groups, there was no excess of major adverse events (eg, heart failure, myocardial infarction, stroke, falls, or transport accidents) or pica. Increasing the frequency of donation did not result in increases in fainting events at donation sessions (table 3). However, the prevalence of self-reported symptoms during the trial period (eg, feeling faint, tiredness, breathlessness, dizziness, and restless legs, especially among men [for all symptoms]) was moderately greater in groups randomly assigned to higher donation frequencies than in the standard frequency groups (all p<0·0001 for men; figure 3 and appendix). Nevertheless, there was no clear evidence of differences across randomised groups in post-hoc analyses investigating more severe self-reported symptoms at 2 years, such as severe shortness of breath (assessed by the MRC Breathlessness Scale^20^) or diagnosed restless legs syndrome (Cambridge-Hopkins questionnaire;^21^ appendix).

**Table 3:** Selected secondary outcomes by sex and intervention group

	**Men**				**Women**			
	8 weeks	10 weeks	12 weeks	p value*	12 weeks	14 weeks	16 weeks	p value*
SF-36v2 mental component score	53·9 (53·8–54·1)	53·9 (53·7–54·0)	53·9 (53·7–54·0)	0·590	52·5 (52·3–52·7)	52·6 (52·5–52·8)	52·6 (52·4–52·8)	0·655
Physical activity energy expenditure, kJ/kg per day†	53·8 (52·6–55·0)	52·3 (51·2–53·4)	52·5 (51·3–53·6)	0·143	39·5 (38·7–40·4)	40·5 (39·5–41·4)	40·1 (39·2–41·0)	0·176
Fainting at donation session (%)‡	0·32 (0·27–0·38)	0·31 (0·26–0·37)	0·32 (0·25–0·38)	0·848	0·78 (0·68–0·88)	0·79 (0·68–0·89)	0·91 (0·79–1·03)	0·139
Serious adverse events (%)§	4·44 (3·94–4·94)	4·01 (3·53–4·49)	4·20 (3·71–4·69)	0·462	4·50 (3·99–5·00)	4·43 (3·93–4·93)	4·48 (3·98–4·99)	0·954
Deferral for low haemoglobin (%)‡	5·72 (5·50–5·95)	3·74 (3·54–3·94)	2·56 (2·38–2·74)	<0·0001	7·92 (7·61–8·24)	6·63 (6·31–6·94)	5·07 (4·78–5·35)	<0·0001
Deferral for other reasons (%)‡	4·34 (4·16–4·52)	4·54 (4·35–4·74)	4·77 (4·55–4·98)	0·004	6·51 (6·25–6·77)	6·91 (6·63–7·19)	7·21 (6·91–7·51)	0·001
Haemoglobin concentration at 2 years, g/L	143·1 (142·7–143·4)	144·7 (144·4–145·0)	146·4 (146·1–146·7)	<0·0001	130·7 (130·4–131·0)	131·5 (131·2–131·8)	132·2 (131·9–132·4)	<0·0001
Haemoglobin concentration at 2 years <135 g/L (men) or <125 g/L (women) (%)¶	18·02 (16·88–19·17)	14·03 (13·02–15·04)	10·25 (9·40–11·11)	<0·0001	19·39 (18·17–20·62)	18·50 (17·33–19·67)	15·65 (14·57–16·73)	<0·0001
Ferritin concentration at 2 years, μg/L||	25·7 (24·9–26·5)	31·1 (30·2–31·9)	36·3 (35·4–37·2)	<0·0001	21·9 (21·2–22·5)	23·3 (22·7–24·0)	26·0 (25·3–26·7)	<0·0001
Ferritin concentration at 2 years <15 μg/L (%)¶||	23·78 (22·07–25·48)	17·61 (16·17–19·05)	12·12 (10·94–13·30)	<0·0001	26·64 (24·85–28·42)	26·43 (24·70–28·16)	21·77 (20·19–23·36)	<0·0001

Data presented are mean or % (95% CI). Missing data (beyond the 0·5% withdrawing permission to use their data): none for deferrals and fainting, 32·0% for 2-year haemoglobin concentration, 35·6% for mental component score, 41·5% for physical activity energy expenditure, 59·2% for 2-year ferritin concentration. Higher mental component scores indicate better mental wellbeing (0–100 scale range). SF-36v2=36-item Short Form Health Survey, version 2.

* p values are for linear trend across groups, from analyses adjusted for baseline characteristics (centre, age, weight, and new donor status) and baseline measurements (where available).

† Assessed with recent physical activity questionnaire.

‡ Per donation session attended, averaged over all attendances.

§ Percentage of participants reporting any serious adverse events over 2 years, in any of the 6-monthly questionnaires, including doctor-confirmed heart failure, heart attack, angina, stroke, or transient ischaemic attack; or hospital visit for falls or transport accidents.

¶ Among those donating blood at 2 years.

|| Results of the 2-year ferritin data are post-hoc analyses (ie, they were not prespecified in the published statistical analysis plan).

The availability of haemoglobin data (recorded for 30 645 [68·0%] of 45 042 participants) was similar across randomised groups. Mean haemoglobin concentrations at 2 years were lower, and the proportion of participants with haemoglobin concentrations below the minimum regulatory threshold was higher, in donors allocated to shorter intervals than in those allocated to the standard donation intervals (p<0·0001; table 3 and appendix). The rate of deferral for low haemoglobin per session attended (table 3; appendix) increased in men from 2·56% in the 12-week group to 5·72% in the 8-week group (p<0·0001). For women, corresponding rates were 5·07% in the 16-week group and 7·92% in the 12-week group (p<0·0001). The number of men deferred for low haemoglobin at least once during the 2-year trial period was 836 (12%) of 6958 in the 12-week group, 1386 (20%) of 7030 in the 10-week group, and 2303 (33%) of 7074 in the 8-week group; the number of women deferred was 1202 (18%) of 6774 in the 16-week group, 1685 (24%) of 6955 in the 14-week group, and 2199 (31%) of 6994 in the 12-week group. In a non-randomised comparison restricted to donors with at least a 2-year history of donation before trial enrolment, the deferral rate for low haemoglobin among men per session attended in the 12-week group was 2·5 times greater (2·7% *vs* 1·1%) during the trial than in the 2 years before the study (appendix). For women in the 16-week group, the corresponding rate was 1·3 times greater (4·8% *vs* 3·8%) during the trial than in the 2 years before the study (appendix).

The availability of ferritin data was similar across randomised groups (appendix). There were no major differences in the baseline characteristics of the randomly selected subset of 18 392 participants with ferritin data compared with other study participants who provided 2-year samples (appendix). Mean ferritin concentrations at 2 years were lower in donors allocated to shorter intervals than in those allocated to the standard frequency group (p<0·0001). The numbers of men donating blood at 2 years with ferritin concentrations less than 15 μg/L were 359 (12%) of 2952 in the 12-week group, 484 (18%) of 2737 in the 10-week group, and 598 (24%) of 2525 in the 8-week group. For women, the corresponding numbers were 558 (22%) of 2572 in the 16-week group, 643 (26%) of 2470 in the 14-week group, and 655 (27%) of 2419 in the 12-week group (table 3). An observational post-hoc analysis within randomised groups at the 2-year examination suggested modest associations between haemoglobin or ferritin concentrations and symptoms linked with increased frequency of donation (appendix). The randomised effects of increased donation frequency on self-reported symptoms attenuated nearly uniformly, but only slightly, after adjustment for haemoglobin or ferritin concentrations, or both, measured at the 2-year examination (appendix).

**Figure 4:**
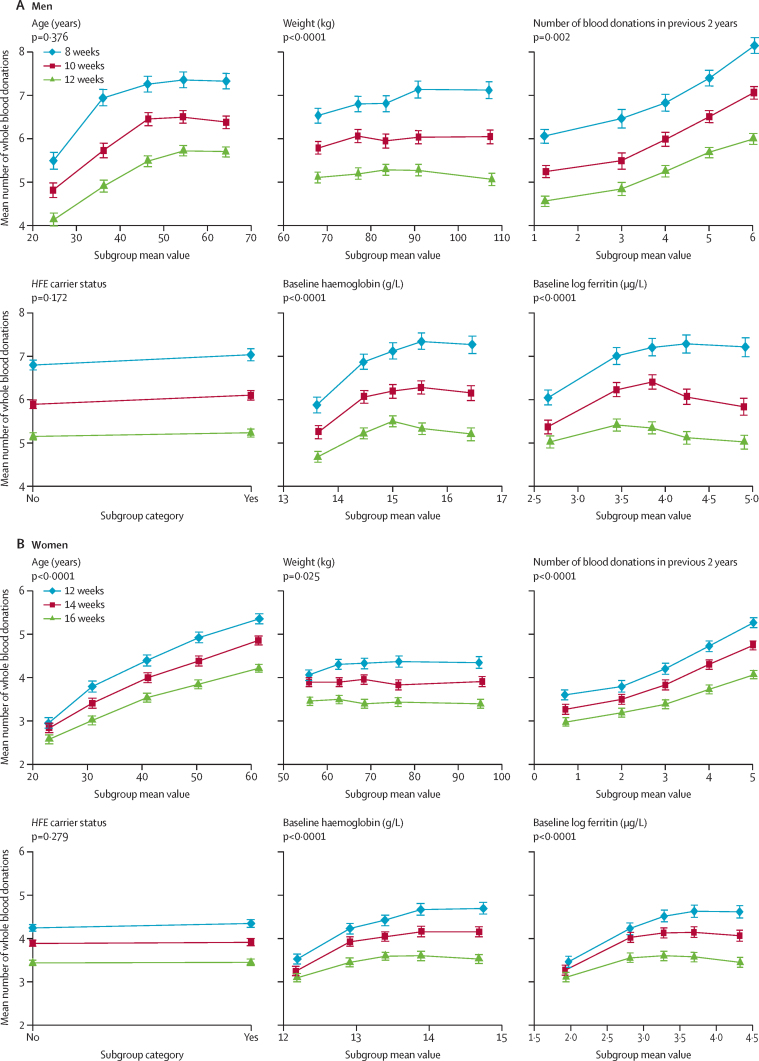
Number of whole blood donations in prespecified subgroups Mean (95% CI) numbers of whole blood donations during the 2-year trial period are shown for men (A) and women (B). Continuous baseline variables are presented in quintile groups. p values correspond to tests for continuous interaction with randomised group. Analysis by ferritin concentration was not prespecified.

In prespecified subgroup analyses (figure 4), in men the mean difference in the number of donations over 2 years across the randomised groups increased with increasing weight and with baseline haemoglobin and ferritin concentrations (all p<0·0001). For women, the mean difference in the number of donations increased with age, the number of donations given in the 2 years before the trial, and baseline haemoglobin and ferritin concentrations (all p<0·0001). There was no evidence of interaction of *HFE* carrier status and randomised groups in relation to amount of blood collected or physical component score at 2 years (appendix; all p>0·1). The statistical significance of the interactions mentioned above persisted in multivariable models including all the interactions (data not shown).

## Discussion

Our trial's main outcome was that, over a 2-year period, there was a substantial increase in the amount of blood collected by reducing the inter-donation blood donation intervals used in the UK to those used in blood services in the USA or western Europe. For example, reducing the inter-donation interval from 12 weeks to 8 weeks in men led to an increase of 33% (1·7 units), and reducing the interval from 16 weeks to 12 weeks in women led to an increase of 24% (0·8 units). Furthermore, prespecified subgroup analyses suggested that even greater proportional gains in blood collection could be achieved by focusing, for frequent donation, on donors with higher than average weight or on those with higher than average initial haemoglobin or ferritin concentrations.

By contrast, the trial's safety findings were more nuanced. On the one hand, the trial showed that reducing inter-donation intervals did not have a major adverse effect on quality of life (assessed with SF-36v2, the most widely used and validated instrument available^15^), cognitive function, or physical activity. On the other hand, reducing inter-donation intervals resulted in greater self-reporting of symptoms potentially related to blood donation, such as tiredness, feeling faint, breathlessness, dizziness, restless legs, and palpitations, especially among men. Because the modest increases we observed in these symptoms were not captured by the results on quality of life, SF-36v2 might not have sufficient sensitivity for minor morbidity in our study's context.^22^, ^23^ This interpretation is consistent with our results showing that reducing inter-donation intervals did not result in more severe morbidity. For example, we found that reducing inter-donation intervals did not result in more severe degrees of breathlessness, diagnosis of restless legs syndrome, the need for medical attention related to palpitations, actual fainting events, or the other major adverse events we recorded.

With regard to biochemical endpoints, we found that reducing inter-donation intervals resulted in decreased mean haemoglobin and serum ferritin concentrations. Whereas the absolute decreases in mean haemoglobin concentrations were modest (around 1–2%) at the 2-year examination, they were large for serum ferritin (around 15–30%), reflecting the increased sensitivity of serum ferritin compared with that of haemoglobin as an indicator of body iron stores.^24^ In particular, our data provide convincing evidence of the cumulative effect of donating blood frequently on haemoglobin concentrations and iron stores. For example, almost a third of both male and female donors allocated to the trial's shortest donation intervals (ie, 8 weeks for men and 12 weeks for women) were deferred for low haemoglobin at least once during the 2-year trial period. Deferrals are essential to protect donors, but they are time consuming and costly for blood services and demotivating for donors.^25^, ^26^

Similarly, by the end of the trial period, about a quarter of both male and female donors allocated to the shortest donation intervals had depleted iron stores according to WHO criteria.^19^ Perhaps surprisingly, however, we found that serum ferritin and haemoglobin concentrations explained only a small part of the symptoms linked with increased frequency of donation. Previous trials of iron supplementation have not reported clear or substantial improvement in such symptoms (eg, fatigue or restless legs), even among people with iron deficiency anaemia.^27^, ^28^ Hence, the results of our trial will invite further study on this matter.^29^

Our data could have several potential implications for blood donation practice and policy. First, our data give policy makers in the UK the short-term option of allowing more frequent collection from donors than is now standard, such as for in-demand blood groups or during periods of falling supply. A priority, therefore, is to identify which donors best tolerate more frequent donation to operationalise this policy.^30^ Second, our data quantify the extent of iron depletion within 2 years of repeated donation, which could inform safety guidelines for blood services that allow more frequent donation than in the UK (eg, the USA, France, and Germany). Canadian Blood Services have recently lengthened the inter-donation interval for women from 8 weeks to 12 weeks.^31^ The US Food and Drug Administration and the AABB (formerly the American Association of Blood Banks) have considered lengthening the 8-week minimum inter-donation interval that currently applies to men and women in the USA to reduce the risk of iron deficiency.^32^, ^33^ Our data highlight the absence of randomised evidence for the long-term safety of frequent donation beyond a 2-year period.

Third, the results of our study found that about 10% of participants were allowed to donate despite having baseline haemoglobin concentrations below the minimum regulatory threshold, suggesting a need to review the screening method used in the UK to test donors' eligibility to donate.^34^, ^35^ (Our study could make this assessment because the trial used a haematology analyser in addition to the copper sulphate test and spectrometry method used in routine eligibility checks.) In response to these findings, we have started the COMPARE study (ISRCTN90871183) to provide a systematic, within-person comparison of the relative merits of different haemoglobin screening methods.

Fourth, our findings underscore the potential benefits of effective communication with blood donors about making and keeping donation appointments. Indeed, we found that gains in blood collection achieved through comprehensive reminders were similar to those obtained by reducing inter-donation intervals^36^, ^37^ (although part of this gain is likely to have been due to the effects of participating in a research study^38^). Finally, as this trial has identified subsets of donors with readily measured characteristics (eg, those with higher than average ferritin concentrations) who have greater capacity than other donors to give blood more frequently, our study contributes to the possibility of increased personalisation of blood donation.

Our trial had major strengths. Because it was the first randomised trial of the effect of varying the frequency of whole blood donation, it provides uniquely reliable insight compared with observational studies that are liable to confounding.^9^, ^10^, ^11^ The trial recorded a range of outcomes related to efficiency, safety, and biochemistry (achieved through linkage with donor health records, validated self-report instruments, and objective measurements), making it more comprehensive and valid than previous observational studies. Because most donors attended blood donation appointments according to the inter-donation intervals allocated, the trial achieved a clear separation between randomised groups. As the trial was embedded in the routine blood service, it achieved rapid recruitment across the geographical breadth of England.^13^ More than 45 000 participants were recruited and randomly assigned, providing excellent statistical power to compare three sex-specific inter-donation intervals. Since we had access to the blood service's national database, the trial achieved 99·5% completeness for the primary outcome. Finally, internet-based approaches enabled frequent and efficient data collection and in-built quality checks.

The study also had potential limitations. Only about two-thirds of participants responded to the 2-year questionnaire. However, material bias because of selective response seems unlikely because questionnaire response rates were nearly uniform across randomised groups; moreover, the results were similar to those from an analysis of 6-monthly interim questionnaires that included 86% of all participants. Absence of blinding (which was not possible because of the nature of the intervention) could explain some of the increased frequency of self-reported symptoms among participants allocated to more frequent donation than the standard practice. However, any such effects could not have influenced the outcomes we assessed through laboratory assays or linkage with donor health records. At the 2-year final survey, the link between measures of iron depletion and symptoms associated with more frequent donation could have been weakened because there was potential dissociation in time between symptoms (questionnaires asked about symptoms experienced at any time in the previous 6 months) and haemoglobin and ferritin concentrations (which were measured in samples collected at the 2-year survey). Our previous analysis had suggested that participants in the INTERVAL study were broadly representative of the national donor population of England.^13^ However, the exact degree of generalisability is uncertain because only about 45% of donors who were invited and attended donation sessions agreed to participate in the trial, and because the trial required participants to have internet access.

In summary, during a period of 2 years, more frequent donation than is standard practice in the UK led to collection of substantially more blood without having a major effect on donors' quality of life, physical activity, or cognitive function. However, this approach resulted in more donation-related symptoms, deferrals, and iron deficiency. Our study has provided the first randomised evaluation, including precise quantification, of key measures of efficiency and safety that blood services need to balance to safeguard donor health and maintain the blood supply.
