# Supplementary material for: Efficiency and safety of varying the frequency of whole blood donation (INTERVAL): a randomised trial of 45 000 donors
Source: Lancet. 2017 Nov 25;390(10110):2360–71. doi: 10.1016/S0140-6736(17)31928-1 (PMC5714430; doi:10.1016/S0140-6736(17)31928-1)
Supplement: Supplementary appendix [file mmc1.pdf]

THE LANCET

## Supplementary appendix

This appendix formed part of the original submission and has been peer reviewed. We post it as supplied by the authors.

Supplement to: Di Angelantonio E, Thompson SG, Kaptoge S, et al, on behalf of the INTERVAL Trial Group. Efficiency and safety of varying the frequency of whole blood donation (INTERVAL): a randomised trial of 45 000 donors. *Lancet* 2017; published online Sept 20. http://dx.doi.org/10.1016/S0140-6736(17)31928-1.

**Supplementary Appendix**

Efficiency and safety of varying the frequency of whole blood donation:

randomised trial of 45,000 donors

**Pages**


| | |
|---|---|
| 2 | List of investigators |
| 5 | Supplementary Methods |
| 8 | Table S1: Characteristics of NHSBT general donor population and of the INTERVAL trial. |
| 9 | Table S2: Cognitive function tests at 2 years after randomisation |
| 10 | Table S3: Reported pica over 2 years, and self-reported symptoms on 6-monthly questionnaires. |
| 11 | Table S4: Post-hoc analysis of other 2-year outcomes. |
| 12 | Table S5: Baseline characteristics of donors with and without information on the 2-year ferritin level |
| 13 | Table S6: Mean haemoglobin levels (g/L) in participants with and without symptoms at 2-year examination |
| 14 | Table S7: Mean ferritin levels (ug/L) in participants with and without symptoms at 2-year examination |
| 15 | Table S8: Analysis of haemoglobin and ferritin as potential mediators of the intervention effect on reported symptoms at 2-year examination |
| 16 | Figure S1: CONSORT flow chart |
| 17 | Figure S2: Adherence to randomised donation intervals |
| 18 | Figure S3: Number of whole blood donations during the 2-year trial period (primary outcome) restricted to donors with at least a 2-year history of blood donation before trial enrolment |
| 19 | Figure S4: Mean Physical Component Score at 2 years and at baseline |
| 20 | Figure S5: Percentage of donors with low haemoglobin level at 2 years and at baseline, restricted to those meeting usual whole blood donation criteria at 2 years |
| 21 | Figure S6: Deferral rates for low haemoglobin level restricted to donors with at least a 2-year history of blood donation before trial enrolment. |
| 22 | Figure S7: Pre-specified subgroup analyses for key secondary outcome |

**List of investigators**

**The INTERVAL Trial Group members**

*Writing Committee (Manuscript Authors):* Emanuele Di Angelantonio MD PhD*, University of Cambridge and NHS Blood and Transplant; Simon G Thompson FMedSci*, University of Cambridge; Stephen Kaptoge PhD*, University of Cambridge; Carmel Moore PhD*, University of Cambridge; Matthew Walker PhD, University of Cambridge; Jane Armitage FRCP FFPH, University of Oxford; Willem H Ouwehand FMedSci, University of Cambridge and NHS Blood and Transplant; David J Roberts DPhil FRCPath†, University of Oxford and NHS Blood and Transplant; John Danesh FMedSci†, University of Cambridge.

*Joint first authors. †Joint last authors.

*Trial Steering Committee*: Jane Armitage FRCP FFPH (independent chair), University of Oxford; John Danesh FMedSci, University of Cambridge; Emanuele Di Angelantonio MD PhD, University of Cambridge and NHS Blood and Transplant; Jenny Donovan FMedSci (independent member), University of Bristol; Ian Ford FRSE FRCP (independent member), University of Glasgow; Rachel Henry, University of Cambridge; Beverley J Hunt FRCP, FRCPath (independent member), King's College, London; Bridget le Huray (lay member); Susan Mehenny, NHS Blood and Transplant; Gail Miflin FRCP FRCPath, NHS Blood and Transplant; Carmel Moore PhD, University of Cambridge; Willem H Ouwehand FMedSci, University of Cambridge and NHS Blood and Transplant; Jane Green, NHS Blood and Transplant; David J Roberts DPhil FRCPath, University of Oxford and NHS Blood and Transplant; Mike Stredder, NHS Blood and Transplant; Simon G Thompson FMedSci, University of Cambridge; Matthew Walker PhD, University of Cambridge; Nicholas A Watkins PhD, NHS Blood and Transplant. Previousmembers: Alan McDermott, NHS Blood and Transplant; Clive Ronaldson, NHS Blood and Transplant; Claire Thomson MSc, University of Cambridge; Zoe Tolkien PhD, University of Cambridge; Lorna Williamson FRCP, NHS Blood and Transplant.

*Trial Management Committee*: David Allen DPhil, University of Oxford and NHS Blood and Transplant; John Danesh FMedSci, University of Cambridge; Emanuele Di Angelantonio MD PhD, University of Cambridge and NHS Blood and Transplant; Rachel Henry, University of Cambridge; Susan Mehenny, NHS Blood and Transplant; Carmel Moore PhD, University of Cambridge; Willem H Ouwehand FMedSci, University of Cambridge and NHS Blood and Transplant; David J Roberts DPhil FRCPath, University of Oxford and NHS Blood and Transplant; Jennifer Sambrook PhD, University of Cambridge; Matthew Walker PhD, University of Cambridge. Previous members: Tracey Hammerton, NHS Blood and Transplant; Claire Thomson MSc, University of Cambridge; Zoe Tolkien PhD, University of Cambridge.

*Haematology Review Group*: David Allen DPhil, University of Oxford and NHS Blood and Transplant; David Bruce MBChB FRCPath, Oxford University Hospitals NHS Foundation Trust; Fizzah Choudry MRCP, University of Cambridge; Emanuele Di Angelantonio MD PhD, University of Cambridge and NHS Blood and Transplant; Cedric Ghevaert MD PhD FRCP FRCPath, University of Cambridge; Kirstie Johnston, NHS Blood and Transplant; Anne Kelly MB BChir PhD, University of Cambridge; Andrew King BM BCh MRCP FRCPath, Weatherall Institute of Molecular Medicine, University of Oxford; Susan Mehenny, NHS Blood and Transplant; Gail Miflin FRCP FRCPath, NHS Blood and Transplant; Alfred Mo MRCGP, NHS Blood and Transplant; Carmel Moore PhD, University of Cambridge; Willem H Ouwehand (co-chair) FMedSci, University of Cambridge and NHS Blood and Transplant; Lizanne Page LMSSA, NHS Blood and Transplant; Penny Richardson, NHS Blood and Transplant; David J Roberts (co-chair) DPhil FRCPath, University of Oxford and NHS Blood and Transplant; Jennifer Sambrook PhD, University of Cambridge; Peter Senior, NHS Blood and Transplant; Yagnesh Umrania PhD, University of Cambridge; Matthew Walker PhD, University of Cambridge; Henna Wong MBBS MRCP FRCPath, Oxford University Hospitals NHS Foundation Trust.

*Restless legs syndrome adviser*: Brendan Burchell PhD, University of Cambridge.

*Cognitive function assessment adviser*: John Gallacher PhD FFPH, University of Oxford and University of Cardiff.

*Independent Data Monitoring Committee*: Stephen Kaptoge PhD (independent statistician), University of Cambridge; Gavin Murphy FRCS, University of Leicester, Adrian C Newland FRCP, FRCPath (chair), Barts and The London NHS Trust, Queen Mary's School of Medicine and Dentistry; Keith Wheatley BA, DPhil, University of Birmingham. Previous members: Michael Greaves BChB MD FRCP FRCPath (chair), University of Aberdeen; Marc Turner MBChB MBA PhD FRCP FRCPath, Scottish National Blood Transfusion Service.

*NHSBT Donation Centres (managers)*: Birmingham (Tahir Aziz and Richard Brain); Bradford (Christine Davies and Ruth Turner); Brentwood (Paula Wakeman); Bristol (Alison Dent); Cambridge (Alan Wakeman); Edgware (Ben Anthony, Desmond Bland, and Will Parrondo); Gloucester (Helen Vincent); Lancaster (Candy Weatherill); Leeds CBTU (Andrea Forsyth); Leeds City (Carol Butterfi eld); Leicester (Tracey Wright and Karen Ellis); Liverpool (Kristie Johnston and Pat Poynton); Luton (Carolyn Brooks and Emma Martin); Manchester Norfolk House (Lara Littler and Lindsay Williams); Manchester Plymouth Grove (Donna Blair and Karen Ackerley); Newcastle (Lynn Woods); Nottingham (Sophie Stanley and Gemma Walsh); Oxford (Gayle Franklin and Cheryl Howath); Poole (Sarah Sharpe); Plymouth (Deborah Smith); She eld (Lauren Botham); Southampton (Caroline Williams and Claire Alexander); Stoke (Gareth Sowerbutts and Diane Furnival); Tooting (Michael Thake); West End (Shilpa Patel, Carolyn Roost, and Sandra Sowerby).

*NHSBT collaborators*: Mary Joy Appleton, Eileen Bays, Geo- Bowyer, Steven Clarkson, Stuart Halson, Kate Holmes, Gareth Humphreys, Kristie Johnston, Lee Parvin-Cooper, Jason Towler.

*NHSBT INTERVAL Study Administration Team (ISAT)*: Joanne Addy, Patricia Barrass, Louise Stennett.

*INTERVAL Helpdesk*: Susan Burton, Hannah Dingwall, Rachel Henry (previous members Victoria Clarke, Maria Potton, Claire Thomson).

*Trial Data Management Team*: Thomas Bolton, Michael Daynes, Stuart Halson, Sarah Spackman, Michael Walker (previous members Abudu Momodu).

*UK Biocentre*: James Fenton, Adam King, Omer Muhammad, Nicholas Oates, Tim Peakman, Christine Ryan, Kristian Spreckley, Craig Stubbins, Joanna Williams (previous members James Brennan, Cedric Mochon, Samantha Taylor, Kimberly Warren).

*Trial statisticians*: Stephen Kaptoge PhD, University of Cambridge; Simon G Thompson FMedSci, University of Cambridge.

*Co-investigators*: Emanuele Di Angelantonio MD PhD, University of Cambridge and NHS Blood and Transplant; Carmel Moore PhD, University of Cambridge; Jonathan Mant FFPH, FRCPE, University of Cambridge; Willem H Ouwehand FMedSci, University of Cambridge and NHS Blood and Transplant; Simon G Thompson FMedSci, University of Cambridge.

*Chief Investigators*: John Danesh FMedSci, University of Cambridge; David J Roberts DPhil FRCPath, University of Oxford and NHS Blood and Transplant.

*Acknowledgments*

The trial was funded by NHS Blood and Transplant (NHSBT) and the National Institute for Health Research (NIHR) Blood and Transplant Research Unit in Donor Health and Genomics (NIHR BTRU-2014-10024). The trial's coordinating centre at the Department of Public Health and Primary Care at the University of Cambridge, Cambridge, UK, has received core support from the UK Medical Research Council (G0800270), British Heart Foundation (SP/09/002), and

the NIHR (Cambridge Biomedical Research Centre). Investigators at the University of Oxford, Oxford, UK, have been supported by the Research and Development Programme of NHSBT, the NHSBT Howard Ostin Trust Fund, and the NIHR (Oxford Biomedical Research Centre) through the programme grant NIHR-RP-PG-0310-1004 and the Oxford Biomedical Research Centre. We thank the blood donors who participated in the trial and NHSBT's operational staff. We are indebted to NHSBT's chief executive (Ian Trenholm [current] and Linda Hamlyn [previous]) and leadership team (in alphabetical order): Alan McDermott, Jane Green, Gail Miflin, Clive Ronaldson, Mike Stredder, Nicholas A Watkins, and Lorna Williamson.

## SUPPLEMENTARY METHODS

### Sample size

This trial was powered on its primary endpoint (number of blood donations over 2 years) and key secondary endpoint (physical component quality of life score).

(1) Number of blood donations: As the men in this trial were assigned to 12-wk versus 10-wk versus 8-wk inter-donation intervals, the maximum number of donations possible over the 2-year trial duration was 8, 10, and 12 donations, respectively (that is, a 25% proportional increase in donation rates when comparing a 10-wk versus 12-wk frequency, or a 50% proportional increase when comparing a 8-wk versus 12-wk frequency). Women were assigned to 16-wk versus 14-wk versus 12-wk inter-donation intervals, corresponding to a maximum number of donations over 2 years of 6, 7, and 8 respectively. However, when considering the nonattendance rates of donors at the time of trial design, such maximal differences based on more frequent donation intervals seemed unlikely to be achieved in practice. Hence, the power calculations were based on having 80% power to detect a more realistic 5% increase in the number of donations over two years. Such an increase was judged to be of potential relevance to NHSBT since it would yield an additional 70,000 units of blood per year from the same donor base. Inclusion of about 50,000 trial participants was estimated to provide 80% power to detect ≥5% difference in donation rates/year in subgroups with a prevalence of ≥10% (at least 5,000 donors). The sample size calculation assumed type I error = 0.05 and mean donation rate = 1.6 times/yr in the standard donation frequency group (control), and between subject SD = 0.7 times/yr in each group.

(2) Quality of life: After making allowances for up to one third of participants not completing the final questionnaire, inclusion of about 50,000 trial participants was estimated to provide 80% power to detect ≥3% mean difference in the PCS of the SF-36 in subgroups with a prevalence of ≥10%. This assumed a type I error = 0.05 and mean PCS = 50 in the standard donation frequency group (control), and between subject SD = 10 in each group.

### Procedures to encourage donation attendance

The trial used a more comprehensive approach than that routinely used by NHSBT to remind participants to make and keep donation appointments. First, the trial's reminder approach

applied to all participants rather than, as in routine NHSBT practice, a subset of donors judged to be high priority by various criteria (eg, blood group, reliability, sex, recent contact). Second, participants in the trial who had not made an initial donation appointment within 14 days of randomisation received up to three reminders using a fixed protocol of telephone and/or email messages. Third, when a booking had not been made for a subsequent appointment three weeks prior to its due date, participants received an email reminder to book an appointment. Fourth, all participants received a text message reminder one day in advance of each appointment. Fifth, after failure to attend an appointment, participants were encouraged to make new appointments using the process described above for making an initial appointment after randomisation into the trial.

### Deferral period for low haemoglobin level

In NHSBT, haemoglobin screening is done using a gravimetric method (copper sulphate test), followed by the spectrophotometric Hemocue™ test for those who failed the copper sulphate test. The minimum thresholds to donate are 135g/L for men and 125g/L for women in England. Hence, in routine NHSBT practice and in the INTERVAL trial, male donors with haemoglobin levels between 125-134g/L and females with levels 115-124g/L were deferred for a period of 3 months, while donors with lower haemoglobin levels (<125g/L and <115g/L in men and women respectively) were deferred for a period of 12 months. Donors with three consecutive low haemoglobin results on the spectrophotometric Hemocue™ test were withdraw from blood donation altogether.

### Self-reported symptoms collected during follow-up

At 6, 12 and 18 donors were asked about the presence of the following symptoms that had occurred in the prior six months: fainting or feeling faint, more tired than usual, more breathless than usual, palpitations, dizziness, chest pain, and restless legs. At two years after randomisation, participants received the same questionnaire as described above, again focusing on the prior six months. Participants also received additional questions related to: pica (related to the prior two years), shortness of breath using the MRC breathless scale, and restless legs syndrome using the Cambridge-Hopkins questionnaire.

### Further details of statistical methods

Only the number of donations over 2 years and the PCS at 2 years were considered for subgroup analyses, using tests of interaction, for the following five pre-specified baseline variables: age, weight, donation history, carrier status of haemochromatosis (*HFE*) gene variants, and haemoglobin level (considered as continuous linear terms, when relevant). Some secondary outcomes involved combination of data from multiple donation sessions attended, or multiple questionnaires answered, by each individual. In these cases, robust standard errors were used to allow for the extra uncertainty due to clustering by individual. Because of the number of statistical tests performed, we used the following guidelines for considering the results as providing strong evidence: $P<0.005$ for the main analysis of the number of donations over 2 years and PCS at 2 years; $P<0.0005$ for their interaction tests; and $P<0.0002$ for the tests of trend for the other secondary outcomes. This approach controls the overall false-positive rate to $<0.07$. The main analyses omitted donors with missing data on the relevant outcome (complete-case analysis). In sensitivity analyses to help account for missing data in the PCS at 2 years, a linear mixed model was used incorporating data on PCS from all of the 6-monthly questionnaires. Analyses were done with Stata v13 (StataCorp, College Station, TX).

### Unblinded interim analysis of safety results

On 3 February 2014, the Independent Data Monitoring Committee requested that the trial's Steering Committee review un-blinded analyses of safety results. The conclusion of the analysis was to continue the trial without alteration, apart from the addition to the two-year assessment of further questions (eg, MRC Breathlessness Scale) related to potential donation-related symptoms.

### Responsibility for final content of manuscript and submission

The investigators at the trial's academic coordinating centre had sole access to the trial database, and had final responsibility for data integrity, analysis, and interpretation, as well as manuscript drafting and submission.

**Table S1:** Characteristics of NHSBT general donor population and of the INTERVAL trial.

| | NHSBT general donor population | Randomised in INTERVAL trial |
|---|---|---|
| All donors | 1330506 (100.0%) | 45235 (100.0%)* |
| Sex | | |
| *Male* | 586372 (44.1%) | 22456 (49.6%) |
| *Female* | 744134 (55.9%) | 22779 (50.4%) |
| Age at baseline (years) | 42.3 (15.0) | 43.3 (14.2) |
| Ethnicity | | |
| *White* | 1207593 (90.8%) | 41259 (91.2%) |
| *Asian* | 31829 (2.4%) | 941 (2.1%) |
| *Black African* | 3852 (0.3%) | 105 (0.2%) |
| *Black Caribbean* | 4971 (0.4%) | 271 (0.6%) |
| *Black other* | 743 (0.1%) | 29 (0.1%) |
| *Chinese* | 3831 (0.3%) | 141 (0.3%) |
| *Mixed* | 14838 (1.1%) | 606 (1.3%) |
| *Other* | 3558 (0.3%) | 122 (0.3%) |
| *Unknown* | 59291 (4.5%) | 1761 (3.9%) |
| Blood group | | |
| *A RhD positive (A+)* | 397277 (29.9%) | 13768 (30.4%) |
| *A RhD negative (A-)* | 97450 (7.3%) | 3489 (7.7%) |
| *B RhD positive (B+)* | 102650 (7.7%) | 3877 (8.6%) |
| *B RhD negative (B-)* | 27317 (2.1%) | 993 (2.2%) |
| *O RhD positive (O+)* | 460501 (34.6%) | 16398 (36.3%) |
| *O RhD negative (O-)* | 138349 (10.4%) | 5138 (11.4%) |
| *AB RhD positive (AB+)* | 33817 (2.5%) | 1199 (2.7%) |
| *AB RhD negative (AB-)* | 9550 (0.7%) | 368 (0.8%) |
| *Unknown* | 63595 (4.8%) | 5 (0.0%) |
| Distance to nearest static donor centre (miles)[1] | | |
| *0-4* | 252068 (18.9%) | 27971 (61.8%) |
| *5-9* | 245652 (18.5%) | 9236 (20.4%) |
| *10-29* | 584850 (44.0%) | 6832 (15.1%) |
| *30-59* | 220388 (16.6%) | 730 (1.6%) |
| *60+* | 18045 (1.4%) | 93 (0.2%) |
| *Unknown* | 9503 (0.7%) | 373 (0.8%) |
| Donor status at baseline[2] | | |
| *New* | 288653 (21.7%) | 1147 (2.5%) |
| *Occasional* | 226507 (17.0%) | 7053 (15.6%) |
| *More frequent* | 815346 (61.3%) | 37035 (81.9%) |
| Donations (over last 2 years prior to baseline)[3] | | |
| *All donations* | 2.24 (2.37) | 3.33 (2.00) |
| *Whole blood donations* | 2.08 (1.87) | 3.23 (1.80) |
| *Other donations* | 0.16 (1.64) | 0.10 (1.07) |
| Deferrals (during the 2 years prior to baseline)[4] | | |
| *Deferral for low haemoglobin* | 63071 (4.7%) | 3094 (6.8%) |
| *Any other deferral* | 327747 (24.6%) | 13632 (30.1%) |
| Length of NHSBT donation history at baseline (years)[5] | 8.6 (8.44) | 10.9 (8.4) |
| Venue type attended at baseline[6] | | |
| *Static centre* | 99724 (7.5%) | 45235 (100.0%) |
| *Mobile* | 1230782 (92.5%) | 0 (0.0%) |

Data presented are mean (standard deviation) or number of participants (%) unless otherwise stated.

Published in: Moore C, Bolton T, Walker M, et al. Recruitment and representativeness of blood donors in the INTERVAL randomised trial assessing varying inter-donation intervals. *Trials* 2016; **17**:458.

* the characteristics of 28 donors in the INTERVAL trial could not be identified in the PULSE database due to merged donor records.

[1] The correspondence address (used to calculate "Distance to nearest static donor centre" and derive "Region") was correct at the time the data was extracted from PULSE (Nov 2015). Historical correspondence addresses were not available in PULSE.
[2] "New" has been defined according to the classification used by NHSBT i.e. an individual who has not previously provided a full donation is considered to be a new donor. "Occasional" and "More frequent" have been defined as less than or equal to 2 full donations in the last 5 years and more than 2 full donations in the last 5 years, respectively.
[3] Including donations where volume equals "Normal", "Overweight" or "Missing" (not including donations where volume equals "Empty" or "Underweight").
[4] Deferrals during the 2 years prior to baseline were summarised as counts of people with a deferral for low haemoglobin levels, and more generally deferral for reasons other than low haemoglobin.
[5] Length of donation history with NHSBT at baseline was defined as the period of time between baseline and the minimum of date of registration, date of first attendance (whether successful or not) and date of first successful donation.
[6] For venue type some old venues, that are no longer used, do not have an associated PULSE venue code. In these cases, although venue type was missing, because the only venues that were no longer being used were mobile venues, we have assumed that the venue type was mobile.

**Table S2:** Cognitive function tests at 2 years after randomisation.

| | Men | | | | Women | | | |
|---|---|---|---|---|---|---|---|---|
| Randomised group | 8 weeks | 10 weeks | 12 weeks | P-value[1] | 12 weeks | 14 weeks | 16 weeks | P-value[1] |
| **Cognitive function tests:**[2] | | | | | | | | |
| Reaction time (ms) | 1487 (1474, 1499) | 1488 (1475, 1501) | 1495 (1481, 1508) | 0.792 | 1472 (1459, 1485) | 1472 (1459, 1484) | 1479 (1467, 1492) | 0.821 |
| Attention score (ms) | 450 (439, 461) | 447 (436, 459) | 441 (430, 453) | 0.207 | 421 (410, 431) | 427 (416, 437) | 419 (408, 430) | 0.636 |
| Memory (no. of attempts) | 3.30 (3.21, 3.38) | 3.36 (3.28, 3.44) | 3.39 (3.31, 3.48) | 0.227 | 3.13 (3.06, 3.21) | 3.06 (2.99, 3.13) | 3.14 (3.06, 3.21) | 0.892 |
| Intelligence (no. correct) | 5.22 (5.16, 5.28) | 5.24 (5.18, 5.31) | 5.15 (5.09, 5.21) | 0.146 | 5.08 (5.02, 5.15) | 5.05 (4.99, 5.11) | 5.08 (5.02, 5.14) | 0.950 |
| Executive function score | 19429 (19104, 19754) | 19613 (19283, 19943) | 19750 (19418, 20083) | 0.303 | 18148 (17837, 18459) | 18099 (17776, 18423) | 18523 (18193, 18854) | 0.149 |

Data are mean (95% CI). Missing data: 46.8% to 49.3% for 4 cognitive function tests, 62.3% for attention score

[1] P-values are for linear trend across groups, from analyses adjusted for baseline characteristics (centre, age, weight, new donor status). Because of the number of secondary outcomes, only P<0.0002 provides strong evidence of trends across groups according to the trial's pre-specified analysis plan.

[2] Reaction time and attention score from the Stroop test (lower values indicate better performance); episodic memory from the pairs test (lower values indicate better performance); fluid intelligence from the VNR test (higher values indicate better performance); executive function from the trails test (lower values indicate better performance).

**Table S3:** Reported pica over 2 years, and self-reported symptoms on 6-monthly questionnaires.

| | Men | | | | Women | | | |
|---|---|---|---|---|---|---|---|---|
| Randomised group | 8 weeks | 10 weeks | 12 weeks | P-value[1] | 12 weeks | 14 weeks | 16 weeks | P-value[1] |
| **Reported over 2 years (%)** | | | | | | | | |
| Pica (%) | 1.09 (0.80, 1.38) | 0.65 (0.43, 0.88) | 0.82 (0.56, 1.07) | 0.187 | 1.83 (1.45, 2.22) | 1.69 (1.32, 2.05) | 1.75 (1.37, 2.13) | 0.874 |
| **Reported 6-monthly (%):[2]** | | | | | | | | |
| Fainting or feeling faint (%) | 5.47 (5.07, 5.86) | 4.54 (4.19, 4.90) | 4.12 (3.78, 4.45) | <0.0001 | 9.39 (8.88, 9.91) | 8.65 (8.16, 9.15) | 8.23 (7.74, 8.71) | 0.002 |
| More tired than usual (%) | 19.06 (18.34, 19.78) | 16.35 (15.68, 17.02) | 15.80 (15.13, 16.46) | <0.0001 | 24.88 (24.09, 25.68) | 21.60 (20.86, 22.35) | 22.08 (21.33, 22.84) | <0.0001 |
| More breathless than usual (%) | 6.69 (6.25, 7.14) | 5.51 (5.10, 5.92) | 4.85 (4.47, 5.22) | <0.0001 | 7.60 (7.14, 8.07) | 6.48 (6.06, 6.91) | 6.76 (6.33, 7.20) | 0.008 |
| Palpitations (%) | 5.18 (4.77, 5.59) | 4.75 (4.35, 5.15) | 4.27 (3.90, 4.64) | 0.001 | 10.05 (9.48, 10.62) | 8.72 (8.20, 9.25) | 9.40 (8.84, 9.96) | 0.124 |
| Dizziness (%) | 7.63 (7.15, 8.11) | 6.52 (6.09, 6.96) | 6.08 (5.67, 6.50) | <0.0001 | 13.28 (12.67, 13.90) | 12.38 (11.78, 12.98) | 11.80 (11.20, 12.39) | 0.001 |
| Chest pain (%) | 2.88 (2.60, 3.16) | 2.64 (2.37, 2.92) | 2.45 (2.19, 2.71) | 0.031 | 2.73 (2.45, 3.01) | 2.38 (2.12, 2.64) | 2.85 (2.55, 3.15) | 0.514 |
| Restless legs (%) | 13.06 (12.40, 13.72) | 12.18 (11.54, 12.82) | 11.07 (10.45, 11.68) | <0.0001 | 18.13 (17.36, 18.90) | 17.36 (16.60, 18.13) | 17.22 (16.45, 17.99) | 0.106 |

Data are percentage (95% CI). Missing data: 14.1% for adverse events and symptoms, 36.3% for pica

[1] P-values are for linear trend across groups, from analyses adjusted for baseline characteristics (centre, age, new donor status) and month of follow-up questionnaire for symptoms. Because of the number of secondary outcomes, only P<0.0002 provides strong evidence of trends across groups according to the trial's pre-specified analysis plan.

[2] Percentage of individuals reporting these symptoms at 6-monthly intervals, combining data from all the 6-monthly questionnaires

**Table S4:** Post-hoc analysis of other 2-year outcomes.

| | Men | | | | Women | | | |
|---|---|---|---|---|---|---|---|---|
| Randomised group | 8 weeks | 10 weeks | 12 weeks | P-value[1] | 12 weeks | 14 weeks | 16 weeks | P-value[1] |
| Shortness of breath: MRC breathless scale (%) | 2.42 (1.99, 2.85) | 1.95 (1.56, 2.33) | 2.01 (1.62, 2.40) | 0.188 | 4.12 (3.55, 4.69) | 2.77 (2.30, 3.24) | 3.55 (3.02, 4.09) | 0.118 |
| Restless legs syndrome : Cambridge-Hopkins questionnaire (%) | 4.29 (3.72, 4.86) | 4.44 (3.86, 5.02) | 3.63 (3.11, 4.15) | 0.087 | 7.66 (6.90, 8.42) | 7.79 (7.02, 8.55) | 8.66 (7.85, 9.47) | 0.073 |

Missing data: 36.4% for shortness of breath, 29.0% for restless legs syndrome diagnosis
[1] P-values are for linear trend across groups, from analyses adjusted for baseline characteristics (centre, age, new donor status) and month of follow-up questionnaire for symptoms. Because of the number of secondary outcomes, only P<0.0002 provides strong evidence of trends across groups according to the trial's pre-specified analysis plan.

**Table S5:** Baseline characteristics of donors with and without information on the 2-year ferritin level

| | Men | | Women | |
|---|---|---|---|---|
| | **Unavailable** | **Available** | **Unavailable** | **Available** |
| Number of participants[1] | 6378 | 9410 | 6074 | 8982 |
| Age (years) | 47.6 (13.8) | 47.0 (13.3) | 44.4 (13.8) | 44.0 (13.6) |
| Weight (kg) | 85.0 (14.2) | 85.1 (14.4) | 71.7 (14.7) | 71.2 (14.3) |
| SF-36 Physical component score | 56.7 (4.5) | 56.9 (4.3) | 57.0 (4.6) | 57.0 (4.6) |
| SF-36 Mental component score | 54.9 (5.7) | 54.7 (5.9) | 53.8 (6.4) | 53.9 (6.3) |
| Haemoglobin levels (g/L) | 149.7 (9.8) | 149.4 (9.8) | 134.1 (9.0) | 134.1 (9.1) |
| Haemoglobin levels: < 135 g/L (men) or 125 g/L (women) | 311 (5.0%) | 494 (5.3%) | 756 (12.7%) | 1131 (12.9%) |
| Ferritin levels (µg/L)[2] | 45.0 (27.0-74.0) | 45.0 (27.0-73.0) | 27.0 (15.0-45.0) | 27.0 (15.0-44.0) |
| Ferritin levels < 15 µg/L | 517 (8.6%) | 769 (8.7%) | 1332 (23.5%) | 2020 (24.1%) |
| New donor[3] | 352 (5.5%) | 511 (5.4%) | 446 (7.3%) | 707 (7.9%) |

Data presented are mean (standard deviation) or number of participants (%) unless otherwise stated.

[1] Excluding the 0.5% withdrawing permission to use their data. Additional missing data: None for age, weight, or donation history; SF-36 Physical component score 0.7%, SF-36 Mental component score 0.7%, haemoglobin levels 2.0%, ferritin levels 6.4%.
[2] Values are geometric means and interquartile ranges
[3] An individual who has not previously provided a full blood donation.

**Table S6:** Mean haemoglobin levels (g/dL) in participants with and without symptoms at 2-year examination*

| | Men | | | | Women | | | |
|---|---|---|---|---|---|---|---|---|
| | Symptom absent | Symptom present | | | Symptom absent | Symptom present | | |
| | Mean (95% CI) | Mean (95% CI) | Difference (95% CI) | P value | Mean (95% CI) | Mean (95% CI) | Difference (95% CI) | P value |
| Fainting or feeling faint | 14.45 (14.43, 14.47) | 14.25 (14.17, 14.34) | -0.20 (-0.28, -0.11) | <0.0001 | 13.16 (13.13, 13.18) | 12.96 (12.90, 13.02) | -0.19 (-0.26, -0.13) | <0.0001 |
| Dizziness | 14.45 (14.43, 14.48) | 14.29 (14.22, 14.35) | -0.17 (-0.24, -0.10) | <0.0001 | 13.16 (13.14, 13.18) | 13.00 (12.94, 13.05) | -0.17 (-0.22, -0.11) | <0.0001 |
| More tired than usual | 14.46 (14.44, 14.48) | 14.33 (14.28, 14.38) | -0.13 (-0.19, -0.08) | <0.0001 | 13.17 (13.15, 13.19) | 13.01 (12.96, 13.05) | -0.16 (-0.21, -0.11) | <0.0001 |
| Felt more breathless | 14.45 (14.43, 14.47) | 14.28 (14.18, 14.38) | -0.17 (-0.27, -0.07) | 0.001 | 13.15 (13.13, 13.17) | 12.90 (12.81, 12.99) | -0.25 (-0.34, -0.16) | <0.0001 |
| Palpitations | 14.44 (14.42, 14.46) | 14.43 (14.34, 14.51) | -0.01 (-0.10, 0.07) | 0.760 | 13.14 (13.12, 13.16) | 13.10 (13.04, 13.16) | -0.04 (-0.10, 0.02) | 0.184 |
| Restless legs | 14.46 (14.44, 14.48) | 14.34 (14.29, 14.39) | -0.12 (-0.17, -0.06) | <0.0001 | 13.16 (13.14, 13.18) | 13.06 (13.02, 13.10) | -0.10 (-0.15, -0.05) | <0.001 |

*Reported symptoms in the 6-months prior to the 2-year assessment.

**Table S7:** Mean ferritin levels (ug/L) in participants with and without symptoms at 2-year examination*

| | Men | | | | Women | | | |
|---|---|---|---|---|---|---|---|---|
| | Symptom absent | Symptom present | | | Symptom absent | Symptom present | | |
| | Mean (95% CI) | Mean (95% CI) | Ratio (95% CI) | P value | Mean (95% CI) | Mean (95% CI) | Ratio (95% CI) | P value |
| Fainting or feeling faint | 29.8 (29.2, 30.3) | 27.7 (25.5, 30.1) | 0.93 (0.85, 1.01) | 0.103 | 23.4 (23.0, 23.9) | 20.6 (19.2, 22.0) | 0.88 (0.82, 0.94) | <0.001 |
| Dizziness | 29.8 (29.2, 30.4) | 28.0 (26.3, 29.8) | 0.94 (0.88, 1.00) | 0.066 | 23.5 (23.0, 24.0) | 21.2 (20.1, 22.4) | 0.90 (0.85, 0.96) | 0.001 |
| More tired than usual | 29.8 (29.2, 30.4) | 28.7 (27.3, 30.2) | 0.96 (0.91, 1.02) | 0.168 | 23.5 (23.0, 24.1) | 21.6 (20.6, 22.7) | 0.92 (0.87, 0.97) | 0.001 |
| Felt more breathless | 29.9 (29.3, 30.4) | 25.9 (23.7, 28.4) | 0.87 (0.79, 0.95) | 0.003 | 23.3 (22.8, 23.8) | 20.9 (19.1, 22.9) | 0.90 (0.82, 0.98) | 0.022 |
| Palpitations | 29.6 (29.0, 30.2) | 30.2 (27.8, 32.7) | 1.02 (0.94, 1.11) | 0.649 | 23.1 (22.7, 23.6) | 23.2 (21.8, 24.7) | 1.00 (0.94, 1.07) | 0.909 |
| Restless legs | 30.1 (29.5, 30.8) | 26.9 (25.6, 28.3) | 0.89 (0.85, 0.94) | <0.0001 | 23.5 (23.0, 24.0) | 22.1 (21.1, 23.1) | 0.94 (0.89, 0.99) | 0.013 |

*Reported symptoms in the 6-months prior to the 2-year assessment.

**Table S8**. Analysis of haemoglobin and ferritin as potential mediators of the intervention effect on reported symptoms at 2-year examination*

| | Haemoglobin adjustment | | | | Haemoglobin and ferritin adjustment | | | |
|---|---|---|---|---|---|---|---|---|
| | Men | | Women | | Men | | Women | |
| Symptom \ Adjustment | OR (95% CI) per 2-week shorter interval | P value | OR (95% CI) per 2-week shorter interval | P value | OR (95% CI) per 2-week shorter interval | P value | OR (95% CI) per 2-week shorter interval | P value |
| **Fainting or feeling faint** | **804/13373** | | **1229/12446** | | **446/7623** | | **703/7034** | |
| Unadjusted | 1.19 (1.09, 1.30) | <0.0001 | 1.07 (0.99, 1.15) | 0.078 | 1.15 (1.02, 1.29) | 0.024 | 1.05 (0.96, 1.16) | 0.278 |
| Plus baseline haemoglobin | 1.20 (1.10, 1.31) | <0.0001 | 1.07 (0.99, 1.15) | 0.077 | 1.15 (1.03, 1.30) | 0.017 | 1.06 (0.96, 1.17) | 0.222 |
| Plus baseline log ferritin | - | - | - | - | 1.15 (1.03, 1.30) | 0.017 | 1.06 (0.96, 1.17) | 0.216 |
| Plus 2-year haemoglobin | 1.17 (1.07, 1.28) | <0.001 | 1.06 (0.99, 1.14) | 0.106 | 1.14 (1.01, 1.28) | 0.036 | 1.05 (0.95, 1.16) | 0.305 |
| Plus 2-year log ferritin | - | - | - | - | 1.14 (1.01, 1.29) | 0.030 | 1.05 (0.95, 1.16) | 0.310 |
| **Dizziness** | **1279/13371** | | **1898/12445** | | **743/7622** | | **1078/7034** | |
| Unadjusted | 1.14 (1.06, 1.22) | <0.001 | 1.06 (1.00, 1.12) | 0.061 | 1.18 (1.07, 1.29) | 0.001 | 1.07 (0.99, 1.16) | 0.100 |
| Plus baseline haemoglobin | 1.14 (1.06, 1.23) | <0.001 | 1.06 (1.00, 1.12) | 0.065 | 1.18 (1.08, 1.30) | <0.001 | 1.07 (0.99, 1.16) | 0.085 |
| Plus baseline log ferritin | - | - | - | - | 1.18 (1.08, 1.30) | <0.001 | 1.07 (0.99, 1.16) | 0.083 |
| Plus 2-year haemoglobin | 1.12 (1.05, 1.21) | 0.001 | 1.05 (0.99, 1.12) | 0.101 | 1.17 (1.07, 1.29) | 0.001 | 1.07 (0.98, 1.15) | 0.123 |
| Plus 2-year log ferritin | - | - | - | - | 1.17 (1.07, 1.29) | 0.001 | 1.06 (0.98, 1.15) | 0.138 |
| **More tired than usual** | **2161/13373** | | **2571/12445** | | **1245/7622** | | **1450/7033** | |
| Unadjusted | 1.15 (1.08, 1.21) | <0.0001 | 1.05 (1.00, 1.11) | 0.076 | 1.13 (1.05, 1.22) | 0.001 | 1.08 (1.01, 1.16) | 0.030 |
| Plus baseline haemoglobin | 1.15 (1.09, 1.22) | <0.0001 | 1.05 (1.00, 1.11) | 0.073 | 1.13 (1.05, 1.22) | 0.001 | 1.09 (1.01, 1.17) | 0.023 |
| Plus baseline log ferritin | - | - | - | - | 1.14 (1.05, 1.22) | 0.001 | 1.09 (1.01, 1.17) | 0.020 |
| Plus 2-year haemoglobin | 1.13 (1.07, 1.20) | <0.0001 | 1.04 (0.99, 1.10) | 0.131 | 1.12 (1.04, 1.21) | 0.003 | 1.08 (1.00, 1.16) | 0.041 |
| Plus 2-year log ferritin | - | - | - | - | 1.13 (1.05, 1.22) | 0.001 | 1.08 (1.00, 1.16) | 0.036 |
| **Felt more breathless** | **679/13318** | | **741/12401** | | **386/7591** | | **427/7007** | |
| Unadjusted | 1.31 (1.19, 1.44) | <0.0001 | 1.06 (0.97, 1.16) | 0.205 | 1.35 (1.19, 1.53) | <0.0001 | 1.03 (0.92, 1.17) | 0.590 |
| Plus baseline haemoglobin | 1.31 (1.19, 1.44) | <0.0001 | 1.06 (0.97, 1.17) | 0.188 | 1.35 (1.18, 1.53) | <0.0001 | 1.04 (0.93, 1.18) | 0.488 |
| Plus baseline log ferritin | - | - | - | - | 1.35 (1.19, 1.53) | <0.0001 | 1.05 (0.93, 1.18) | 0.466 |
| Plus 2-year haemoglobin | 1.27 (1.15, 1.40) | <0.0001 | 1.05 (0.96, 1.15) | 0.306 | 1.32 (1.16, 1.50) | <0.0001 | 1.03 (0.91, 1.16) | 0.654 |
| Plus 2-year log ferritin | - | - | - | - | 1.29 (1.14, 1.48) | <0.001 | 1.03 (0.91, 1.16) | 0.668 |
| **Palpitations** | **755/13344** | | **1370/12423** | | **454/7609** | | **758/7016** | |
| Unadjusted | 1.09 (0.99, 1.19) | 0.068 | 1.02 (0.96, 1.10) | 0.513 | 1.07 (0.95, 1.20) | 0.263 | 1.02 (0.93, 1.12) | 0.620 |
| Plus baseline haemoglobin | 1.09 (0.99, 1.19) | 0.069 | 1.02 (0.95, 1.09) | 0.570 | 1.07 (0.95, 1.21) | 0.242 | 1.02 (0.93, 1.12) | 0.637 |
| Plus baseline log ferritin | - | - | - | - | 1.07 (0.95, 1.20) | 0.250 | 1.02 (0.93, 1.12) | 0.658 |
| Plus 2-year haemoglobin | 1.08 (0.99, 1.19) | 0.083 | 1.02 (0.95, 1.09) | 0.599 | 1.07 (0.95, 1.20) | 0.290 | 1.02 (0.93, 1.12) | 0.688 |
| Plus 2-year log ferritin | - | - | - | - | 1.08 (0.95, 1.21) | 0.233 | 1.02 (0.93, 1.12) | 0.674 |
| **Restless legs** | **2001/13238** | | **2677/12320** | | **1132/7543** | | **1475/6967** | |
| Unadjusted | 1.09 (1.02, 1.15) | 0.005 | 1.02 (0.97, 1.08) | 0.363 | 1.09 (1.00, 1.17) | 0.039 | 1.02 (0.95, 1.10) | 0.538 |
| Plus baseline haemoglobin | 1.09 (1.03, 1.15) | 0.004 | 1.03 (0.97, 1.08) | 0.328 | 1.09 (1.01, 1.17) | 0.037 | 1.02 (0.95, 1.10) | 0.548 |
| Plus baseline log ferritin | - | - | - | - | 1.09 (1.01, 1.18) | 0.036 | 1.02 (0.95, 1.10) | 0.538 |
| Plus 2-year haemoglobin | 1.07 (1.01, 1.14) | 0.021 | 1.02 (0.96, 1.07) | 0.531 | 1.08 (1.00, 1.17) | 0.057 | 1.01 (0.94, 1.09) | 0.712 |
| Plus 2-year log ferritin | - | - | - | - | 1.05 (0.97, 1.14) | 0.209 | 1.01 (0.94, 1.08) | 0.812 |

*Reported symptoms in the 6-months prior to the 2-year assessment.

Analyses for each symptom are restricted to a common set of participants with available haemoglobin and ferritin levels at baseline and 24 months.

OR, odds ratio

**Figure S1:** CONSORT flow chart: recruitment, participation and completeness of main and secondary outcomes.

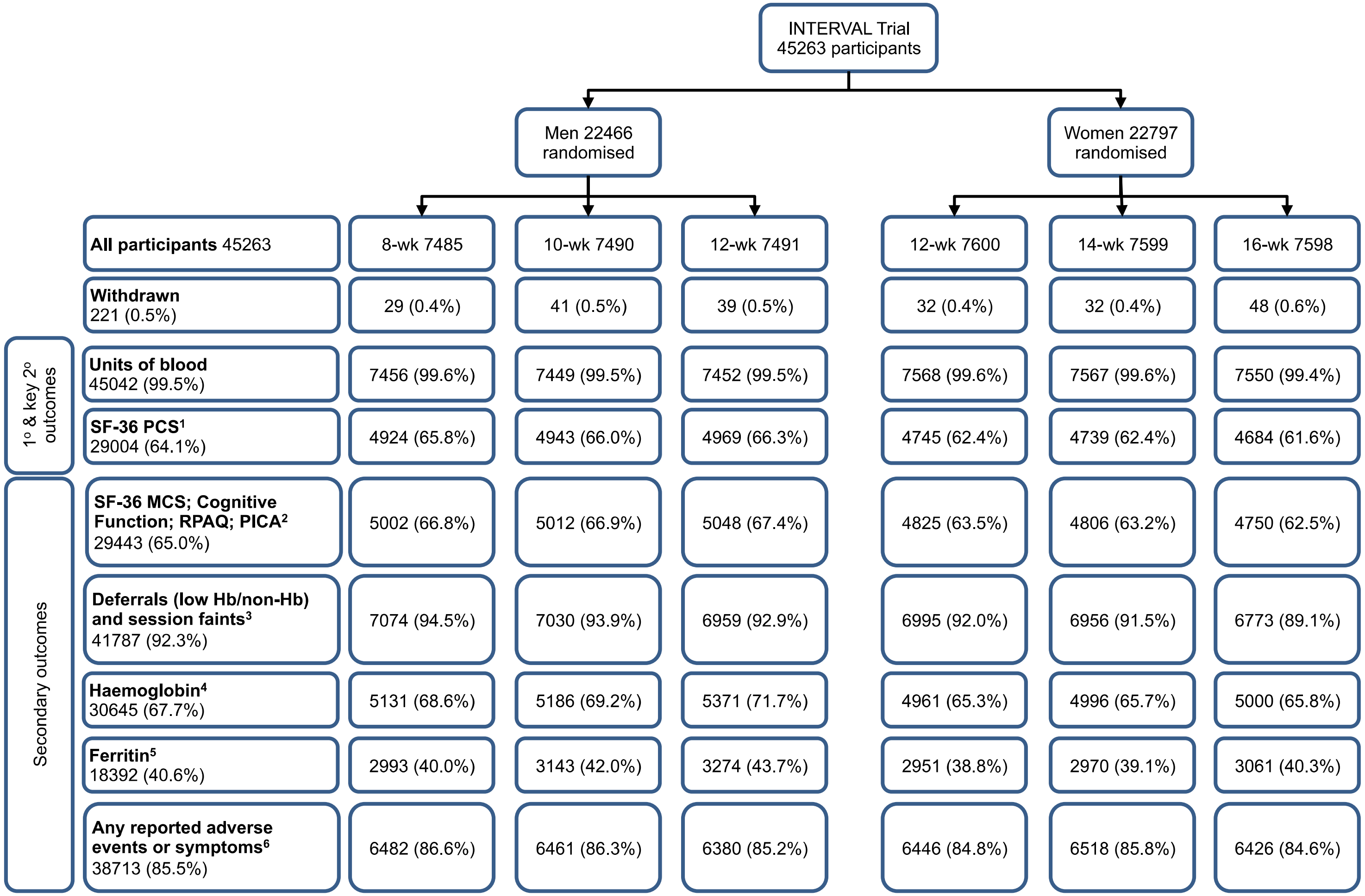


PCS physical component score, MCS mental component score, RPAQ recent physical activity questionnaire, PICA craving for non-food items

[1] number for whom a 2-year PCS score could be calculated

[2] number who have responded to at least one question in the 2-year questionnaire

[3] number who attended at least one donation during their 2-year participation in the trial

[4] number who provided a research blood sample at 2 years on which haemoglobin was measured

[5] number who provided a research blood sample at 2 years on which ferritin was measured and completed by April 2017 (remaining samples are ongoing)

[6] number who responded to at least one question in any of the 6-monthly questionnaires administered during their 2-year participation in the trial, i.e. 6-month, 12-month, 18-month and 2-year questionnaires

**Figure S2:** Adherence to randomised donation intervals (cumulative proportion donating blood or being deferred for low haemoglobin)

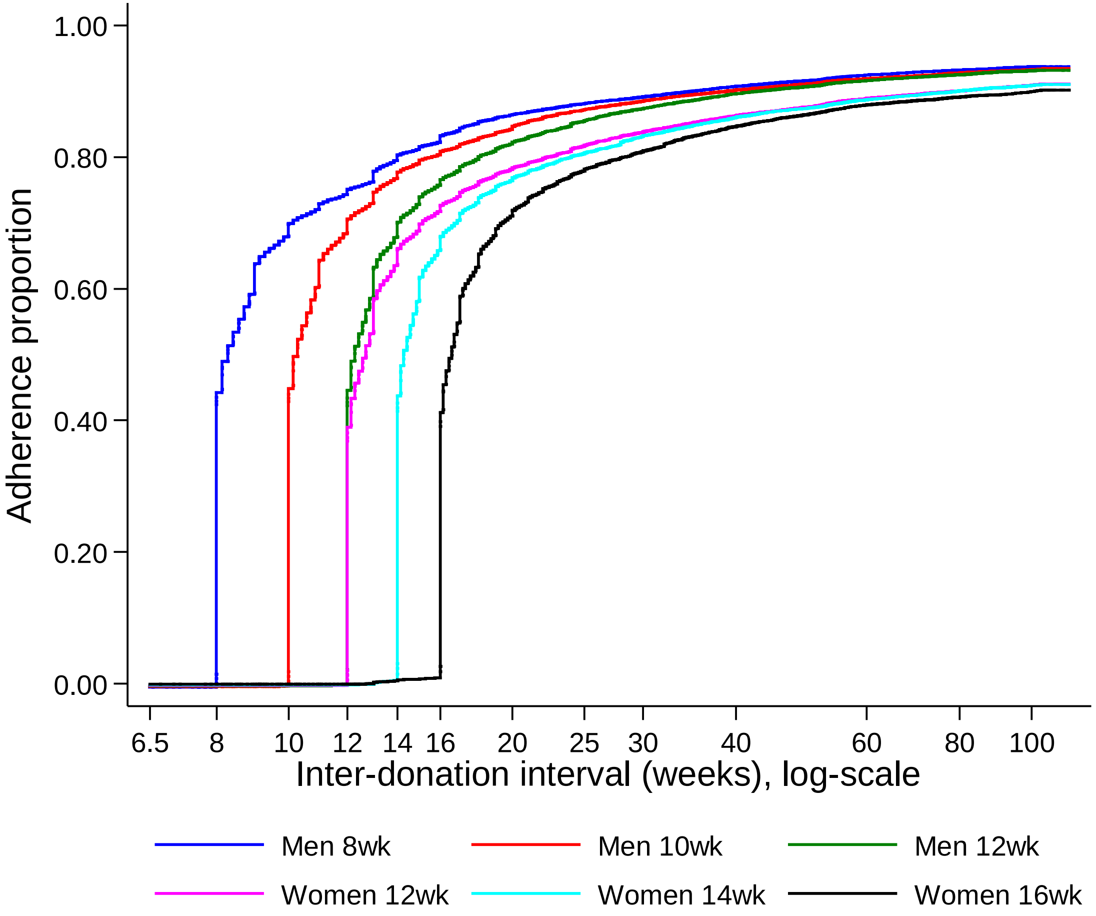

**Figure S3**: Number of whole blood donations during the 2-year trial period (primary outcome) restricted to donors with at least a 2-year history of blood donation before trial enrolment

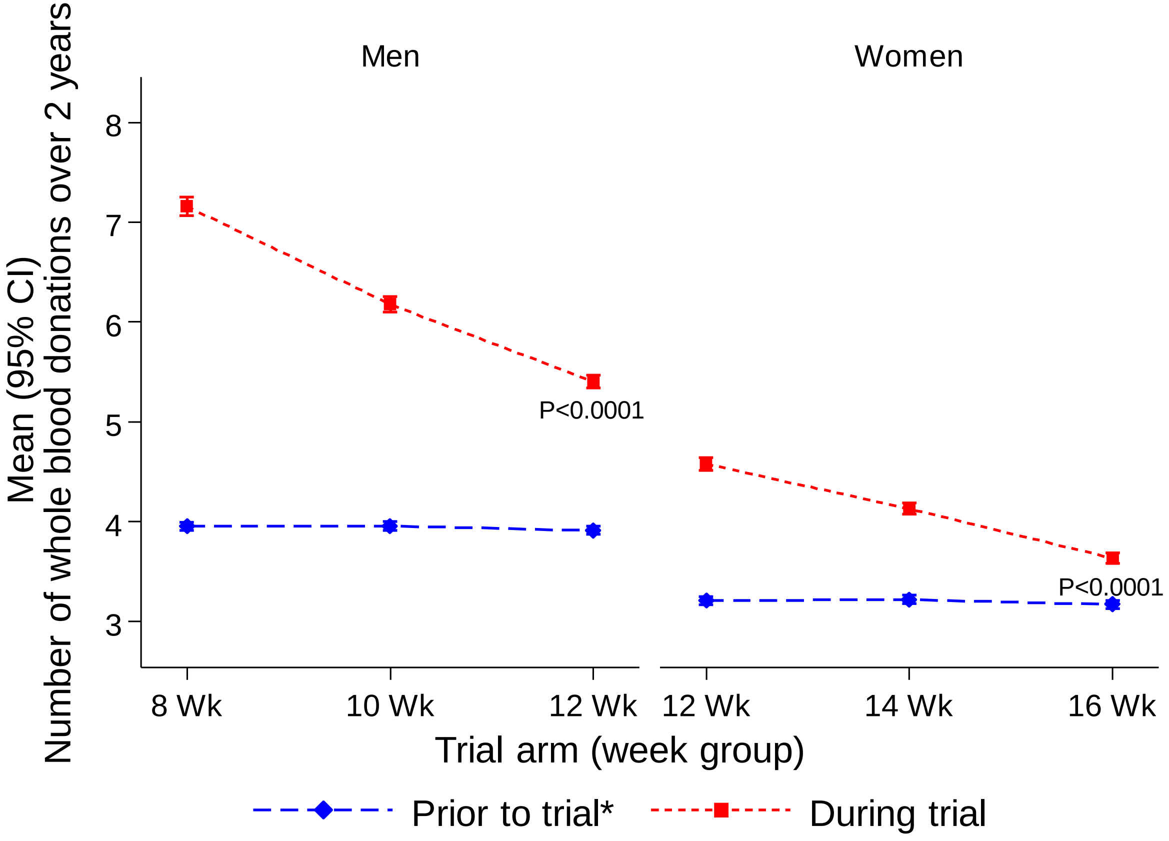


P-values compare randomised groups (red squares).
Baseline values (blue diamonds) are provided purely for context.
*Minimum inter-donation intervals allowed prior to the trial were 12-week for men and 16-week for women.

**Figure S4**: Mean Physical Component Score at 2 years and at baseline by sex and intervention group

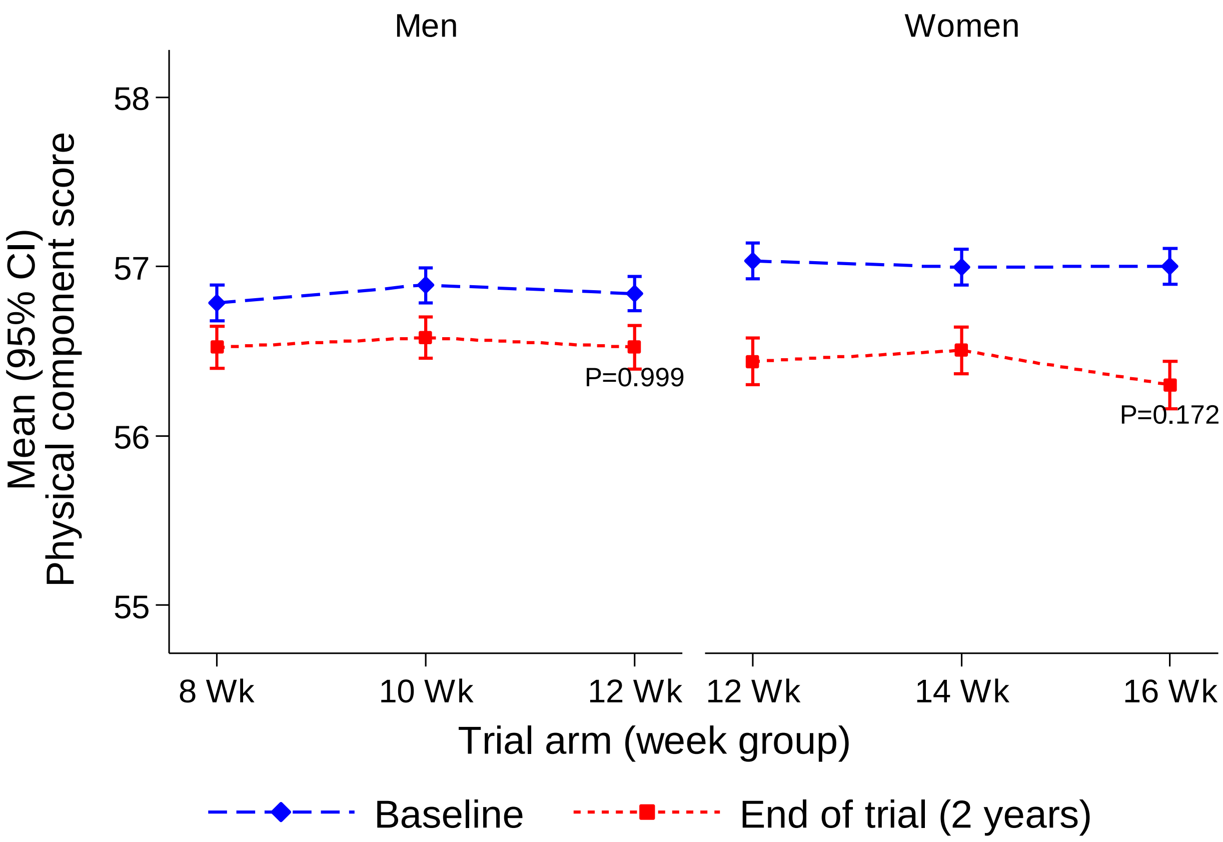


P-values assess trends across randomised groups (red squares).
Baseline values (blue diamonds) are provided purely for context.

**Figure S5**: Percentage of donors with low haemoglobin level (<135 g/L in men and <125 g/L in women) at 2 years and at baseline by sex and intervention group (restricted to those meeting usual whole blood donation criteria at 2 years).

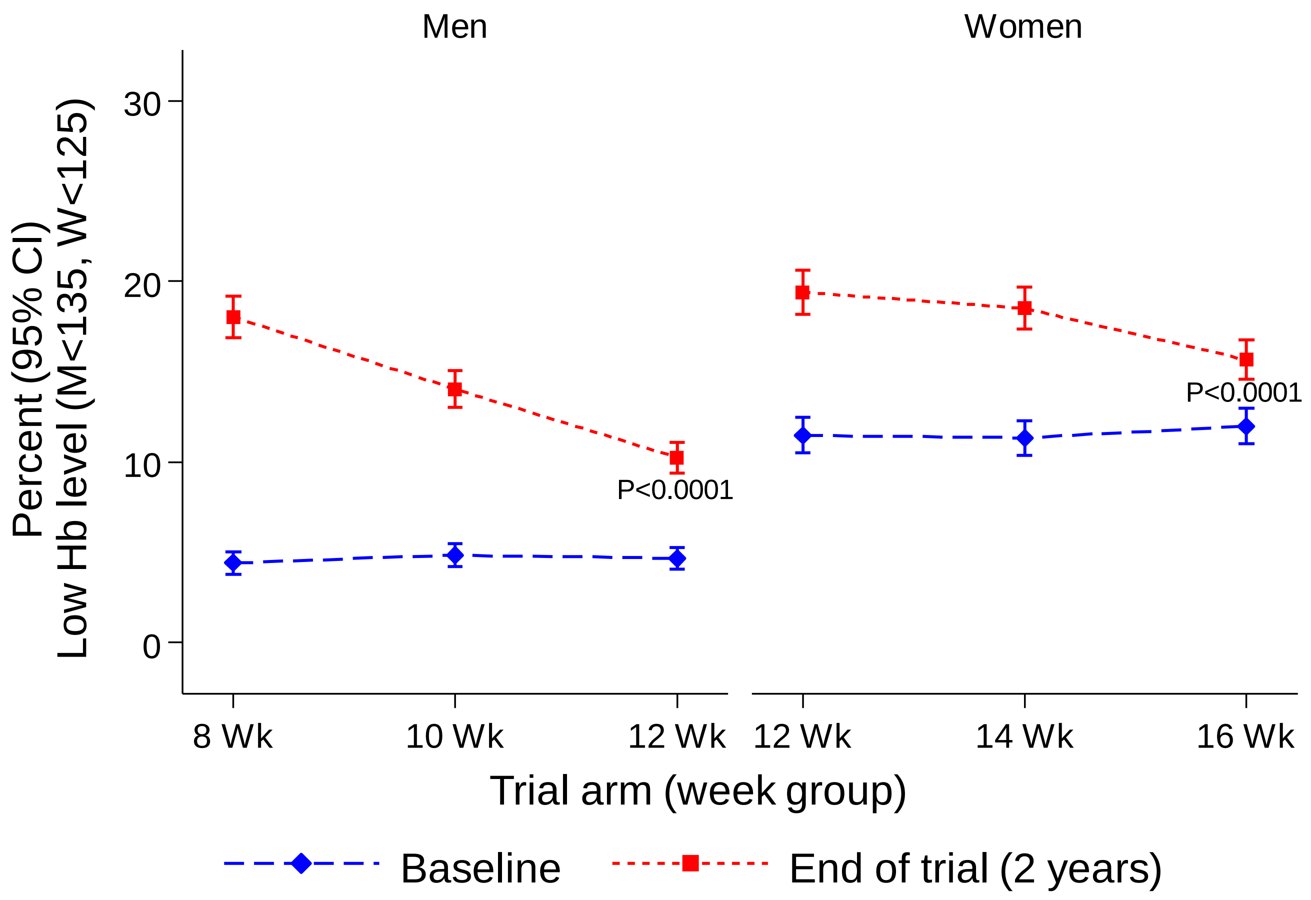


P-values assess trends across randomised groups (red squares).
Baseline values (blue diamonds) are provided purely for context.

**Figure S6**: Deferral rates for low haemoglobin level per donation session attended restricted to donors with at least a 2-year history of blood donation before trial enrolment.

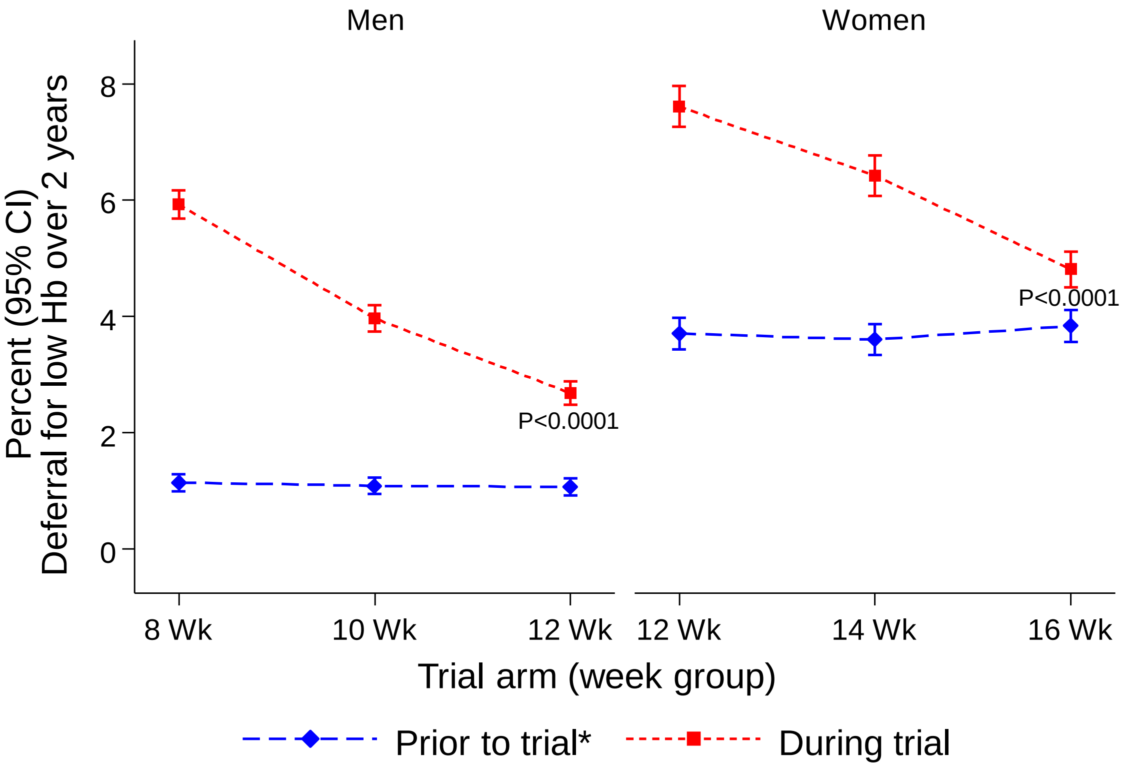


P-values assess trends across randomised groups (red squares).
Baseline values (blue diamonds) are provided purely for context.
*Minimum inter-donation intervals allowed prior to the trial were 12-week for men and 16-week for women.

**Figure S7**: Pre-specified subgroup analyses for key secondary outcome

A) Men

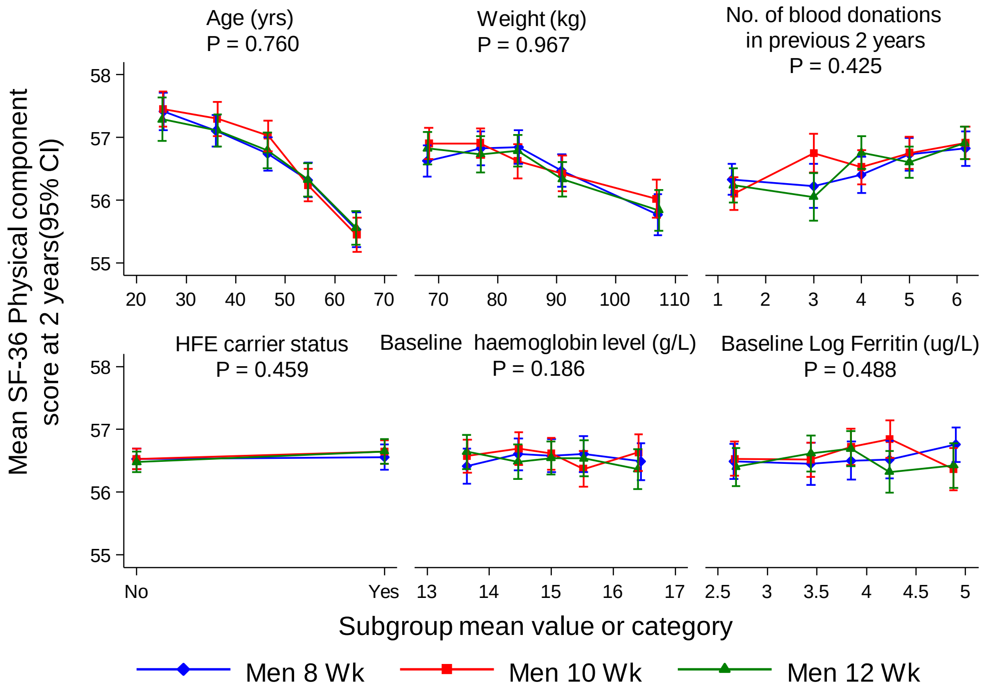


B) Women

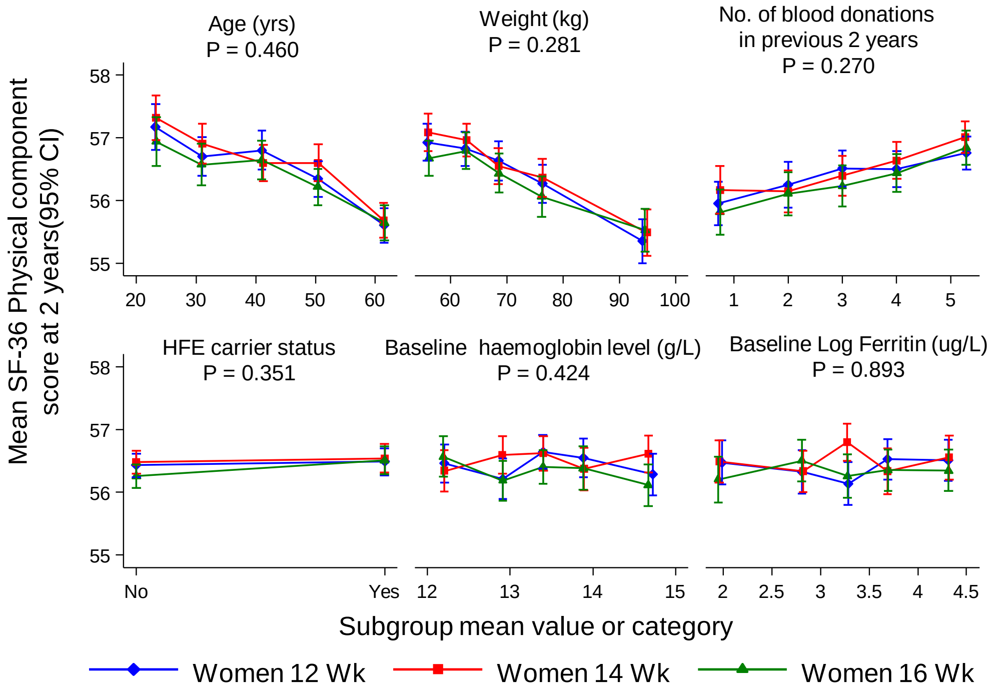


Continuous baseline variable presented in quintile groups
Analysis by ferritin level was not pre-specified.
